# Peripheral blood transcriptome heterogeneity and prognostic potential in lung cancer revealed by RNA‐Seq

**DOI:** 10.1111/jcmm.16773

**Published:** 2021-07-21

**Authors:** Qi Zhang, Manchao Kuang, Haiyin An, Yajing Zhang, Kai Zhang, Lin Feng, Lei Zhang, Shujun Cheng

**Affiliations:** ^1^ State Key Laboratory of Molecular Oncology, Department of Etiology and Carcinogenesis, National Cancer Center/National Clinical Research Center for Cancer/Cancer Hospital Chinese Academy of Medical Sciences and Peking Union Medical College Beijing China; ^2^ Department of Cancer Prevention, National Cancer Center/National Clinical Research Center for Cancer/Cancer Hospital Chinese Academy of Medical Sciences and Peking Union Medical College Beijing China; ^3^ Department of Endoscopy ,National Cancer Center/National Clinical Research Center for Cancer/Cancer Hospital Chinese Academy of Medical Sciences and Peking Union Medical College Beijing China

**Keywords:** heterogeneity, high‐throughput sequencing, lung cancer, peripheral blood leucocytes, prognosis

## Abstract

Understanding of the complex interaction between the peripheral immune system and lung cancer (LC) remains incomplete, limiting patient benefit. Here, we aimed to characterize the host peripheral immune response to LC and investigate its potential prognostic value. Bulk RNA‐sequencing data of peripheral blood leucocytes (PBLs) from healthy volunteers and LC patients (n = 142) were analysed for characterization of host systemic immunity in LC. We observed broad blood transcriptome perturbations in LC patients that were heterogeneous, as two new subtypes were established independent of histology. Functionally, the heterogeneity between the two subtypes included dysregulation of diverse biological processes, such as the cell cycle, blood coagulation and inflammatory signalling pathways, together with the abundance and activity of blood cells, particularly lymphocytes and neutrophils, ultimately manifesting as differences in antitumour immune status. Based on these findings, a prognostic model composed of ten genes dysregulated in one LC subtype with relatively poor immune status was developed and validated in a Gene Expression Omnibus (GEO) data set (n = 108), helping to generate a prognostic nomogram. Collectively, our study provides novel and comprehensive insight into the heterogeneity of the host peripheral immune response to LC. The expression heterogeneity–based predictive model may help guide prognostic management for LC patients.

## 1 INTRODUCTION

The immune response is believed to play a crucial role in the occurrence and progression of cancers, including lung cancer (LC). Past studies have pointed out that, as a highly heterogeneous disease with poor prognosis and high mortality, LC may be promoted via a reprogrammed immune microenvironment by contributing to inflammation, immune regulation and treatment response.^1^, ^2^ Therefore, a variety of diagnostic and prognostic biomarkers have been proposed, and targeted immunotherapy has been applied in clinical therapy based on components of the tumour microenvironment.^3^, ^4^, ^5^ From another perspective, cancer is perceived as a systemic disorder. Immune cell abundance, genomic DNA methylation and serological characteristics have been found to be altered in the peripheral immune system in LC.^6^, ^7^, ^8^ Nevertheless, a comprehensive and in‐depth understanding of the impacts of LC on the host immune system is still lacking.

At present, treatment and prognosis prediction for LC patients are based mainly on the tumour‐node‐metastasis (TNM) staging system.^9^ However, there are still considerable disparities in survival among patients with the same clinical characteristics. Researchers have tried to develop new prognostic auxiliary indicators to enhance the accuracy of LC prognosis,^3^, ^10^, ^11^ but intratumour heterogeneity and sampling bias may limit the validity and reproducibility of prognostic markers, particularly for solid tumour specimens. Hence, reliable prognostic biomarkers are needed to guide adjuvant therapy.

As an accessible source of immune cells that migrate to and from tumour lesions, peripheral blood is exposed to tumour cells and host‐secreted factors released into the peripheral circulation system and is suitable for evaluating the immune status of cancer patients. Furthermore, genome‐wide expression profile analysis based on high‐throughput transcriptome sequencing (RNA‐Seq) of peripheral blood provides an unbiased and in vitro operational approach to measure transcriptional responses in complex diseases, including cancer.^12^, ^13^

In this study, we generated transcriptional profiles of peripheral blood leucocytes (PBLs) from LC patients and healthy subjects by RNA‐Seq. We characterized the transcription profile changes of PBLs in LC and parsed their heterogeneity for the first time. Moreover, we developed a risk score (RS) model with signature genes of PBL subtypes as a good indicator to predict overall survival (OS) for LC patients.

## 2 MATERIALS AND METHODS

See also Supplementary Methods.

### 2.1 Collection of specimens

We recruited 69 healthy individuals and 73 LC patients and collected four millilitres of fresh peripheral blood from each enrolled individual before clinical treatment. The clinical characteristics of all subjects are summarized in Table S1. The study was reviewed and approved by the Ethics Committee of the Cancer Institute and Hospital of the CAMS and was carried out in accordance with the World Medical Association Declaration of Helsinki Ethical Principles for Medical Research.^14^ All subjects provided written informed consent.

### 2.2 Generation and normalization of RNA‐sequencing data

We extracted total RNA from peripheral leucocytes. Eligible libraries were prepared from qualified samples using a NEBNext^®^ Ultra™ RNA Library Prep Kit (New England Biolabs, Ipswich, MA, UK) and sequenced on the Illumina HiSeq 4000 platform. Paired‐end reads (150 bp) were mapped to the human reference genome (GRCh38) by Salmon. Transcript abundances were summarized at the gene level with tximport and were normalized based on transcripts‐per‐million (TPM).

### 2.3 Differentially expressed gene identification and pathway enrichment analysis

The Bioconductor package DESeq2 was used to detect differentially expressed genes (DEGs) with a gene read count matrix.^15^ Only genes with a Benjamini‐Hochberg false discovery rate (FDR) < 0.05 and a |log2 fold change (FC)| ≥ 0.59 were defined as DEGs.

Gene Ontology (GO) and Kyoto Encyclopedia of Genes and Genomes (KEGG) pathway enrichment analyses were performed by the package ClusterProfiler and visualized with the package GOplot and Cytoscape software (v3.7.1) to functionally characterize the identified DEGs.^16^, ^17^ The Benjamini‐Hochberg FDR threshold in each case was set at 0.05.

### 2.4 Estimate the proportion of immune cells

CIBERSORT can differentiate mature human haematopoietic cells by deconvolution with an LM22 signature gene file. To estimate the proportion of 22 immune cell types for each individual, CIBERSORT analysis was performed on the gene expression data with the online tool developed by Newman et al (https://cibersort.stanford.edu/). Routine blood indicators of 67 normal subjects and 37 LC patients collected from medical records were used to verify partial results.

To observe DEGs independent of immune cell composition between LC subtypes, we simplified the cell composition to the ratio of myeloid cells to lymphocytes (myeloid cells include monocytes, M0 macrophages, M1 macrophages, M2 macrophages, resting dendritic cells, activated dendritic cells, resting mast cells, activated mast cells, eosinophils and neutrophils; lymphocytes include naive B cells, memory B cells, plasma cells, CD8 T cells, naive CD4 T cells, memory resting CD4 T cells, memory activated CD4 T cells, T follicular helper cells, regulatory T cells, gamma delta T cells, resting natural killer (NK) cells and activated NK cells) and used it as a covariate in the analysis of differential gene expression.

### 2.5 Dimension reduction and cluster analysis

To explore PBL transcriptional profiling–based heterogeneity among samples, we performed t‐distributed stochastic neighbour embedding (t‐SNE) analysis by the package Rtsne using the first 50 principal components and 1000 iterations, combined with cluster analysis employed by the package mclust with default parameters using all genes.^18^ Principal component analysis (PCA) of all genes and unsupervised hierarchical clustering analysis of the top 3000 most variable genes using the complete method and Pearson correlation distance were carried out by R to validate the gene expression profile heterogeneity between LC subtypes.

### 2.6 Calculation of molecular distance

A metric presented by Pankla et al defined as molecular distance can be applied to quantify global transcriptome variation degree in patients versus normal people.^19^ This method essentially consists of implementing outlier analysis on a gene‐by‐gene basis, where the dispersion of the expression values found in the baseline samples is used to judge whether the expression value of a single case sample lies within two standard deviations of the controls' mean. Here, we calculated the molecular distance of the expression profile for each patient by taking normal samples as the baseline and compared the differences in variation degree between different groups.

### 2.7 Gene set enrichment analysis

To compare differences in the transcriptomes between LC subtypes, we implemented two enrichment methods for the gene expression profiles by taking blood transcription modules (BTMs) devised specifically for blood transcriptome analysis and the ‘hallmark’ MSigDB collection (v7.1) as gene sets: analyses performed with gene set enrichment analysis (GSEA) software (http://www.broad.mit.edu/gsea/) and the tmod package.^20^ Pre‐ranked gene lists consisted of genes arranged with a decreasing metric value derived from the DESeq2 analysis result. The formula used for calculating the metric is as follows:

$$\text{metric}=\frac{‐\log_{10}\left(p\;\text{value}\right)}{\text{sign}(\log_{2}\text{FC})}$$

For tmod, the CERNO test was employed. Only BTMs that were commonly significantly enriched (FDR < 5%) across the two methods were retained.

### 2.8 Weighted gene correlation network analysis

In this study, two gene co‐expression networks consisting of 86 samples (lung cancer set 1 (LC1) vs the normal group) and 125 samples (lung cancer set 2 (LC2) vs the normal group) were established by the package WGCNA.^21^ The two adjacency matrixes were calculated with a beta of 8 or 10, and the minimum cluster size of the clustering dendrograms was 30. The association between eigengene values and clinical traits was assessed by Pearson's correlation, and key co‐expression modules related to class differences were detected according to the correlation and significance *P* values. Genes in each key co‐expression module were ranked by effect size as the mean expression of the LC class minus the expression of the normal class to generate pre‐ranked gene lists for downstream GSEA with BTMs as gene sets.

### 2.9 Construction and validation of a RS prognostic model

We downloaded a transcriptome data set of peripheral blood mononuclear cells (PBMCs) with clinical outcomes from NCBI’s Gene Expression Omnibus (GEO; http://www.ncbi.nlm.nih.gov/geo/) under accession number GSE13255, which contains 108 LC patients. The normalized gene expression matrix transformed by log2 was applied to construct a RS prognostic model. A summary of the sample information is shown in Table S3.

All samples were randomly divided equally into training and testing sets. First, we identified a gene panel selected from LC2 DEGs with *P* < .05 in a univariate regression analysis of the training set (n = 54). Next, we introduced least absolute shrinkage and selection operator (LASSO) Cox regression analysis to the gene panel by the glmnet package.^22^ The expected generalization error was estimated by 10‐fold cross‐validation, and the LASSO model consisting of n genes with non‐zero regression coefficients was determined. Those coefficients (c) and the corresponding gene expression values (E) were used to calculate a RS for each patient, as shown below:

$$RS=c_{1}E_{1}+\cdots c_{n}E_{n}$$

An optimal cut‐off value determined by performing time‐dependent receiver operating characteristic (ROC) analysis with the survival ROC package dichotomized patients into a high‐risk group or a low‐risk group. The Kaplan‐Meier (KM) method with a log‐rank test was used to perform time‐to‐event analysis to evaluate survival differences between the two groups. The testing set and stage I set were utilized to validate the prognostic ability of the predictive model.

Univariate and multivariate Cox proportional hazard regression analyses were performed to assess independent prognostic factors. A novel nomogram was built based on the multivariable analyses and provided visualized risk stratification by the survival, foreign and rms packages. The discrimination of different predictors was appraised by Harrell's concordance index (C‐index).

### 2.10 Statistical analysis

The Mann‐Whitney U test (two‐tailed) was used to compare cell proportions among samples. Pearson's product‐moment correlation test was used to estimate correlations between CIBERSORT analysis results and blood routine indicators of subjects. Fisher's exact chi‐square test, unpaired Student's t test and a chi‐square test with Yates correction for continuity were used to calculate differences in clinical characteristics between different LC subsets. The Kruskal‐Wallis test and Mann‐Whitney U test (two‐tailed) were performed for comparison of molecular distance. All statistical analyses were performed by using R (3.6.3) or SPSS (25.0) software. *P* < .05 was considered statistically significant.

## 3 RESULTS

### 3.1 Broad blood transcriptome perturbations were evident in LC patients

**FIGURE 1:**
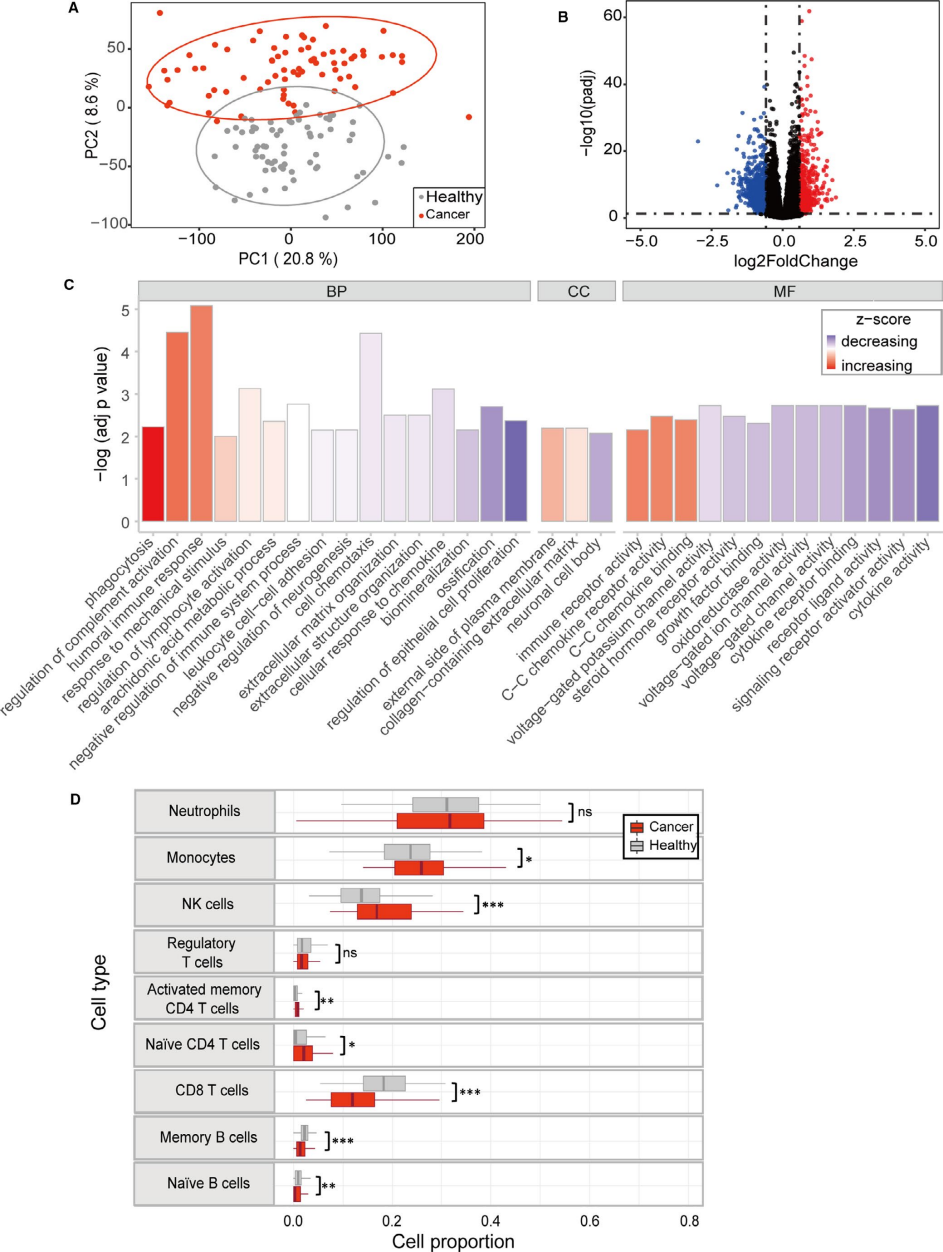
Difference analysis and deconvolution of the PBL transcriptome between LC patients and healthy controls. A, PCA of PBL transcriptional profiles across all subjects. PC1 and PC2 are plotted on the *x*‐axis and *y*‐axis, respectively. Ellipses represent 95% CIs. B, Volcano plot of DEGs in LC versus healthy individuals. The horizontal line indicates FDR = 0.05, and the vertical line indicates |log2FC| = 0.59. Blue, black and red dots represent down‐regulated, non‐significantly differentially expressed and up‐regulated genes, respectively. C, GO enrichment analysis of DEGs (FDR < 0.05) (BP, biology process; CC, cellular component; MF, molecular function). D, Estimated proportions of immune cells in healthy and LC individuals (Mann‐Whitney U test, *P < .05; **P < .01; ***P < .001)

To quantify sample differences in the PBL transcriptome between LC and healthy subjects, we carried out PCA across 142 subjects and found that the LC group was separated from the healthy group (Figure 1A). We identified 1368 DEGs in the LC versus healthy groups to uncover the biological alterations of the peripheral immune system in response to LC (Figure 1B, File S1). Biological processes, including the humoral immune response and regulation of lymphocyte activation, were enriched (Figure 1C). Disturbed pathways identified previously in LC histological studies, such as arachidonic acid metabolism and transcriptional misregulation in cancer, were also observed (Figure S1).^23^, ^24^

We next estimated the proportions of immune cells for each subject by deconvoluting the PBL RNA‐Seq data to investigate changes at the cellular level (File S2). Nine major cell components are displayed (Figure 1D). Changes in the abundance of monocytes, NK cells, T cells and B cells in the patient group were observed, which is in agreement with past research.^25^ Similar changes were found in routine blood indicators that were significantly positively correlated with deconvolution analysis results (Figure S2). In general, the PBL transcriptome of LC patients was widely disturbed and denoted broad aberrancies in innate and adaptive immunity.

### 3.2 PBL transcriptional profiles of LC patients are heterogeneous

**FIGURE 2:**
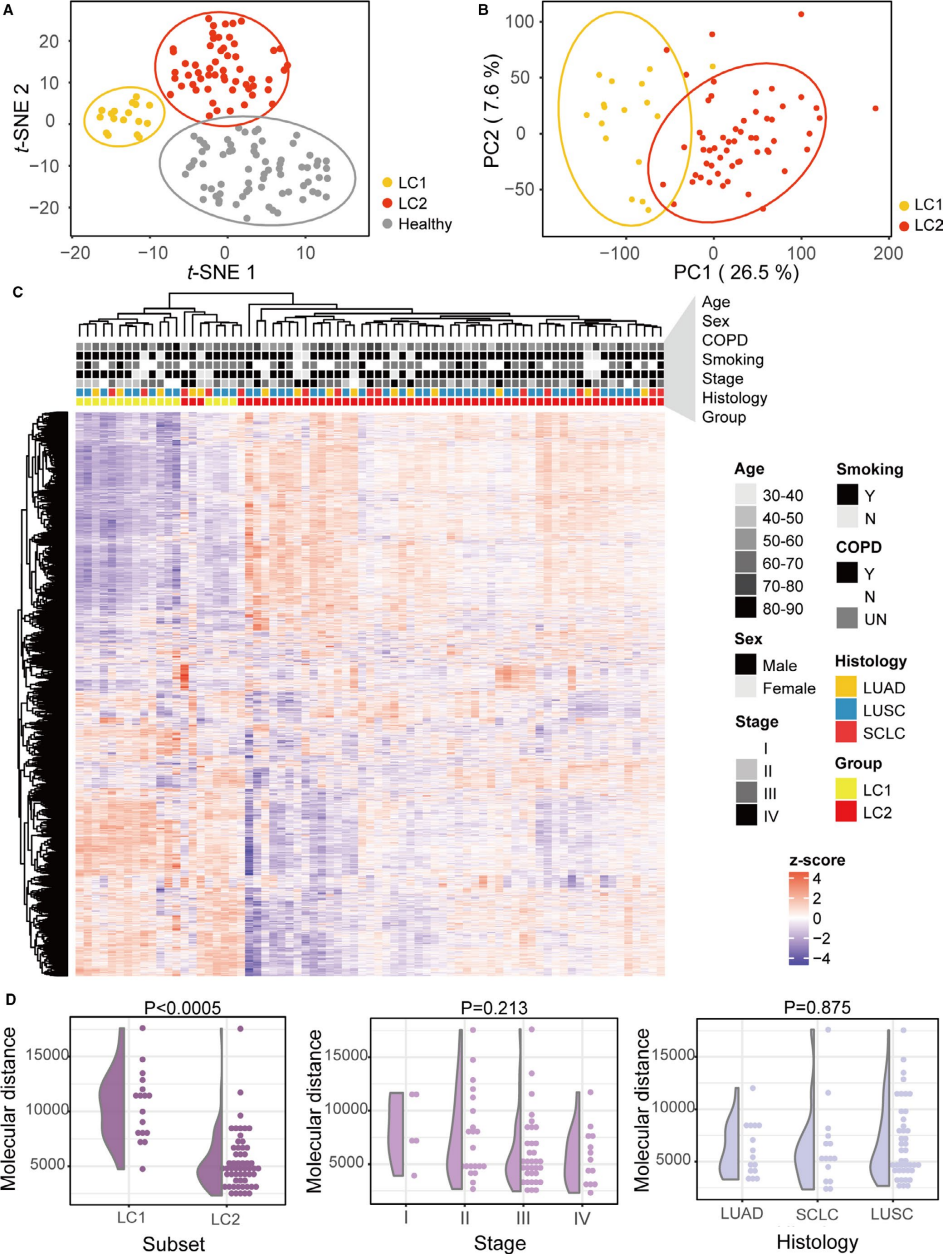
PBL transcriptional heterogeneity in LC. A, t‐SNE plot of RNA‐Seq data of all enrolled samples. Ellipses represent 95% CIs. B, PCA of LC samples. Ellipses represent 95% CIs. C, Unsupervised hierarchical clustering heatmap for the top 3000 most variably expressed genes among LC samples using the complete method and Pearson correlation distance. D, Molecular distance of LC subjects classified by new LC subtypes, TNM stages and histological types (LUAD, lung adenocarcinoma; SCLC, small cell lung cancer; LUSC, lung squamous cell carcinoma). The Mann‐Whitney U test *P* values are displayed on top

We next asked whether there is consistency in peripheral transcriptome perturbations among LC patients. We performed t‐SNE and cluster analyses across all subjects based on the similarity of gene expression profiles (Figure 2A). The LC patients were distinct from normal subjects, in line with the PCA results (Figure 1A). In particular, LC samples were clustered into two subsets defined as LC1 and LC2, both of which contained three LC histological subtypes (Figure S3). We also executed PCA and unsupervised hierarchical cluster analysis of the top 3000 most variable genes to verify between‐group variance (Figure 2B,C). Two clusters were delineated in unsupervised hierarchical cluster analysis, and the vast majority of samples in LC1 and LC2 were divided into different clusters. Detailed clinical traits of the two LC subsets are given in Table S2. Apparently, there were no significant differences in characteristics between the two subsets except for stage (Fisher's exact chi‐square test, *P* = .004). Early‐stage patients were relatively evenly distributed in the two subsets, but more advanced patients were classified as LC2.

In addition, we calculated the molecular distance for each patient to quantify global transcriptional changes in LC over the normal baseline (Figure 2D). Notably, the molecular distance between LC1 and LC2 was significantly different (Mann‐Whitney U test, *P* < .0001). Although the *P* value did not indicate statistical significance (Kruskal‐Wallis test, *P* = .213), there seemed to be a slight difference between subgroups classified based on stage. For the subgroups with different histological types, their PBL expression profiles were very similar (Kruskal‐Wallis test, *P* = .875). These results further imply that the difference in LC histological type with different origins is not manifested in the PBL expression profiles.

In short, considerable heterogeneity of PBL transcriptional profiles independent of histological type was identified in LC. We next conducted analyses aiming to interpret the heterogeneity and its significance.

### 3.3 Global alterations revealed the heterogeneity of the PBL transcriptome in LC patients

**FIGURE 3:**
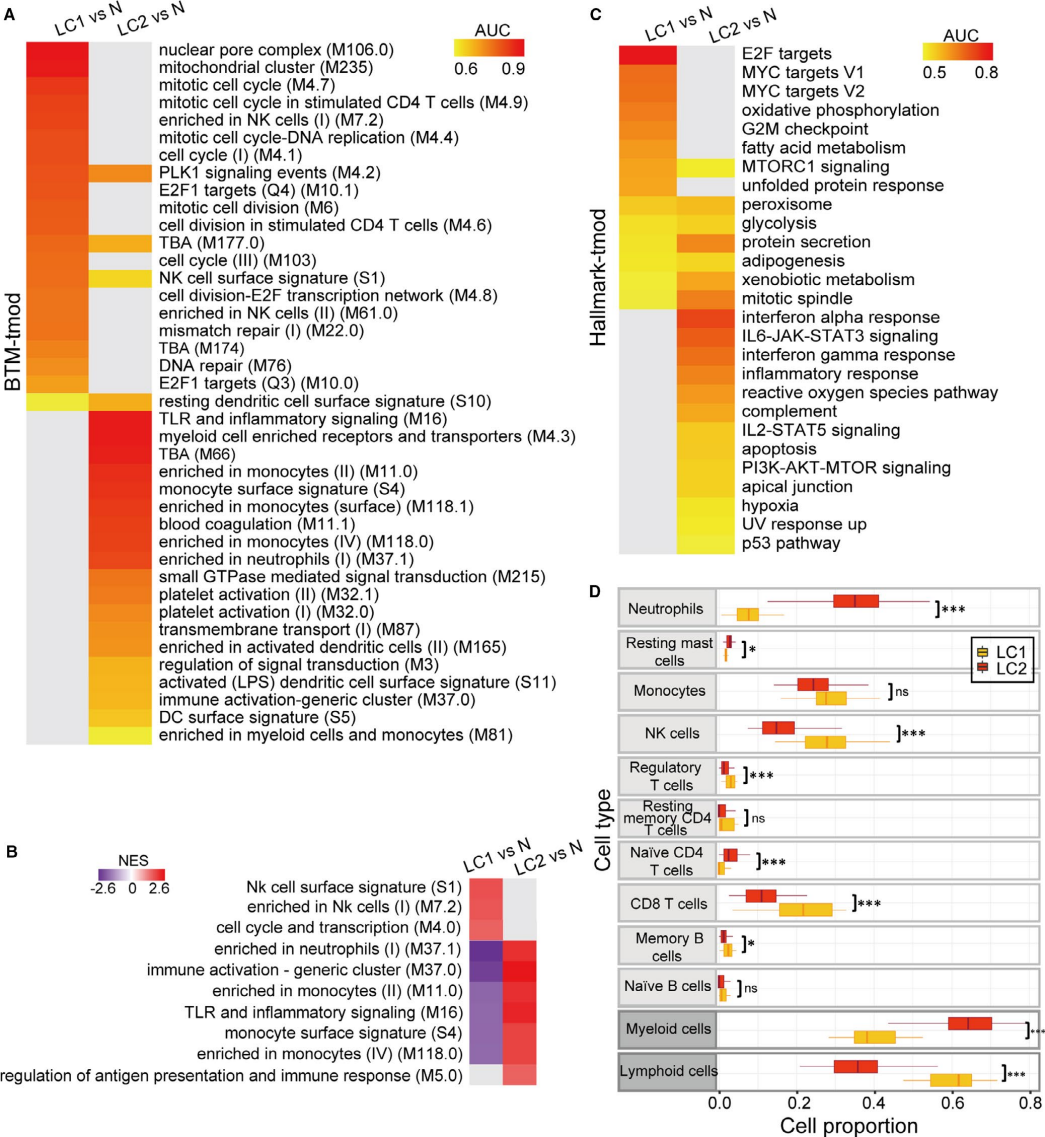
Global diverse PBL transcriptional alterations revealed by gene enrichment analyses. A, BTMs differentially enriched between classes in both GSEA and tmod (FDR < 0.05 across both methods). Only tmod results are shown (N, normal controls). See also Figure S4A. B, BTM enrichment analysis of genes in significant co‐expression network modules constructed by WGCNA based on LC versus normal controls. Key modules of LC1 network include the brown, magenta and turquoise modules. Key modules of LC2 network include the tan, magenta and turquoise modules. Only significant BTMs (FDR < 0.05) are shown. C, Hallmark gene sets from MSigDB v7.1 differentially enriched between classes in two testing methods (FDR < 0.05 across both methods). Only tmod results are shown. See also Figure S4B. D, Comparison of immune cell proportions in each LC subset. (Mann‐Whitney U test; ns, not significant, *P* > .05; *P < .05; **P < .01; ***P < .001)

First, to characterize the global blood transcriptional profile, we explored BTM signatures of the LC subsets devised specifically for blood transcriptome analysis by performing GSEA and tmod analysis. Significantly up‐regulated BTMs in LC1 versus normal controls (N) across the two methods (FDR < 0.05) included NK cell signature enrichment, cell cycle and DNA repair relevant pathways. Up‐regulated BTMs in LC2 included myeloid cell signature enrichment, blood coagulation and inflammatory signalling pathways (Figure 3A and Figure S4A). We next implemented WGCNA to evaluate class differences in the co‐expression network module derived from LC‐induced data (Figure S5). Core modules related to disease states were found to be differentially enriched in the two subsets, most of which were consistent with the above enrichment analyses results (Figure 3B, File S3). Furthermore, hallmark signatures related to the cell cycle, such as E2F targets, MYC targets and the G2M checkpoint, were salient in LC1.^26^, ^27^ LC2 highlighted the inflammatory response and cytokine‐mediated signalling pathway (Figure 3C and Figure S4B).

In view of the enrichment of immune cell signatures, we also focused on different cell abundances between the new LC subtypes. Neutrophils and lymphocytes, especially NK cells and CD8 T cells, showed significant differences between LC1 and LC2 by deconvolution and routine blood analysis (Figure 3D and Figure S6). Moreover, the estimated neutrophil‐to‐lymphocyte ratio of LC2, that is an indicator of the inflammation level, was significantly higher than that of LC1 (1.09 ± 0.55 vs 0.15 ± 0.10).

Overall, heterogeneity in the PBL transcriptome of LC patients involved cell cycle–related pathways, blood coagulation, inflammatory signalling pathways and PBL composition, which may play crucial roles in the LC subsets.

### 3.4 Peripheral antitumour immune status is distinct between the LC subsets

**FIGURE 4:**
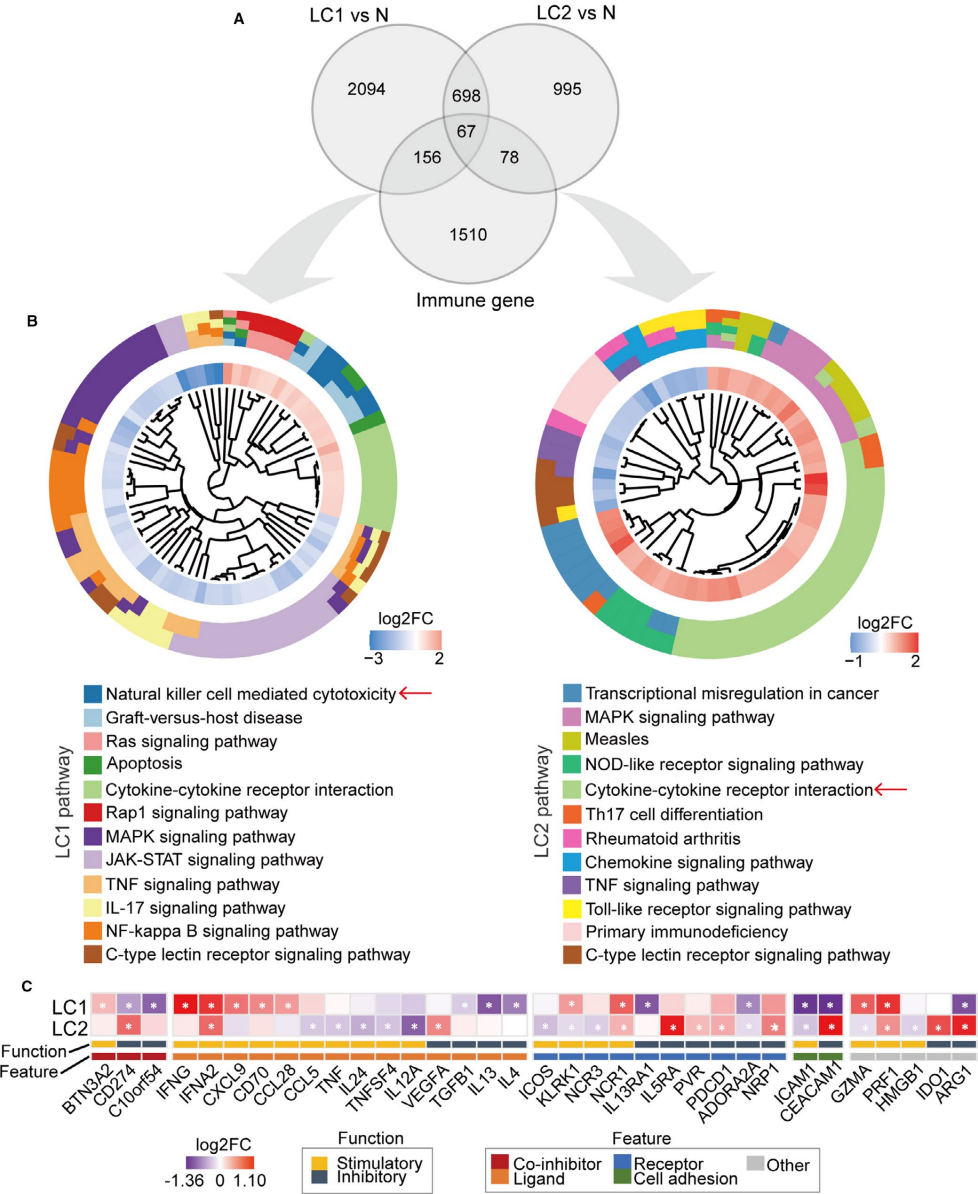
Antitumour immunological differences between the LC subsets. A, Gene overlap between immune genes and two DEG sets was identified by a Venn diagram. B, KEGG pathway enrichment analysis with two panels of immune‐related DEGs showing highly significant pathways (FDR < 0.05) in the two subgroups. Only the top five up‐regulated and down‐regulated pathways are shown in each group. The inner circle shows the log2FC values of DEGs in enriched pathways. C, Expression pattern of immunomodulators (IMs) in LC1 and LC2. Log2FC values are shown as a heatmap. White asterisks indicate genes with differential expression (FDR < 0.05). Colours of annotation bars denote different molecular functions and features

Given the global abnormalities related to immunity of the PBL transcriptome and its leading role in the peripheral immune response, we further assessed differences in immune status between two LC subsets based on DEGs. Two DEG panels from LC1 vs N (3015 DEGs) and LC2 vs N (1838 DEGs) were identified, with only 765 genes overlapping (Figure 4A, File S4). We focused on immunological aberrancies by matching the two DEG sets with a list of immune‐associated genes from ImmPort (https://www.immport.org) (Figure 4A). A total of 223 and 145 overlapping DEGs were enriched in different pathways, including up‐regulated NK cell–mediated cytotoxicity in LC1 and cytokine‐cytokine receptor interaction in LC2 (Figure 4B). Considering the immune cell proportion differences, we identified two DEG signatures that were independent of cell components for the two LC subsets (Figure S7A, File S4). Most of the above enriched pathways remained striking and independent of cell composition, emphasizing the critical role in the immune response to different LC subtypes (Figure S7B).

Immunomodulators (IMs) are pivotal for modulation of the immune response level. To gain insight into the peripheral immune status, we next evaluated the expression of a list of IMs that may stimulate or inhibit the immune response to LC (Figure 4C).^28^ The results showed that the expression of IMs varied across the LC subsets. In LC1, stimulatory IMs, such as *CD70*, *CXCL9* and NK cell activation receptor‐*NKG2D* (*KLRK1*), were up‐regulated.^29^ Comparatively, most immunosuppressive molecules, such as *PD1* (*PDCD1*) and *PDL1* (*CD274*), showed significant up‐regulation, while stimulatory IMs, including *NKG2D*, tended to be down‐regulated in LC2 cells. These findings underscored the possible peripheral immunosuppression in antitumour immunity of LC2 compared to LC1.

### 3.5 Gene signatures of the PBL transcriptome have the capability of predicting LC survival

Remarkable alterations induced by LC in the PBL transcriptome prompted us to determine whether relevant gene signatures have prognostic value for LC. Considering the more advanced status of the patients, the inflammatory and suppressed peripheral immune status in LC2, we speculated that LC2 may have a poorer prognosis and its signature genes have good performance in survival prediction for LC patients. Hence, we took the 1838 DEGs identified from LC2 as candidate genes to develop and validate a prognostic prediction model within a PBMC expression profile data set (n = 108). Eventually, a ten‐gene signature (*HK3*, *SLC36A1*, *MSR1*, *CEACAM1*, *CEACAM6*, *HCG27*, *FXYD7*, *TRPLC1*, *NR3C2* and *RLN2*) predictive model was constructed with univariate regression and LASSO Cox regression analysis (Table S4 and Figure S8A,B,E). Most of the ten genes are expressed in monocytes or lymphoid cells and differentially expressed across the LC subsets in our study, suggesting possible differential outcomes between LC1 and LC2 (https://www.proteinatlas.org/) (Figure S8F).

**FIGURE 5:**
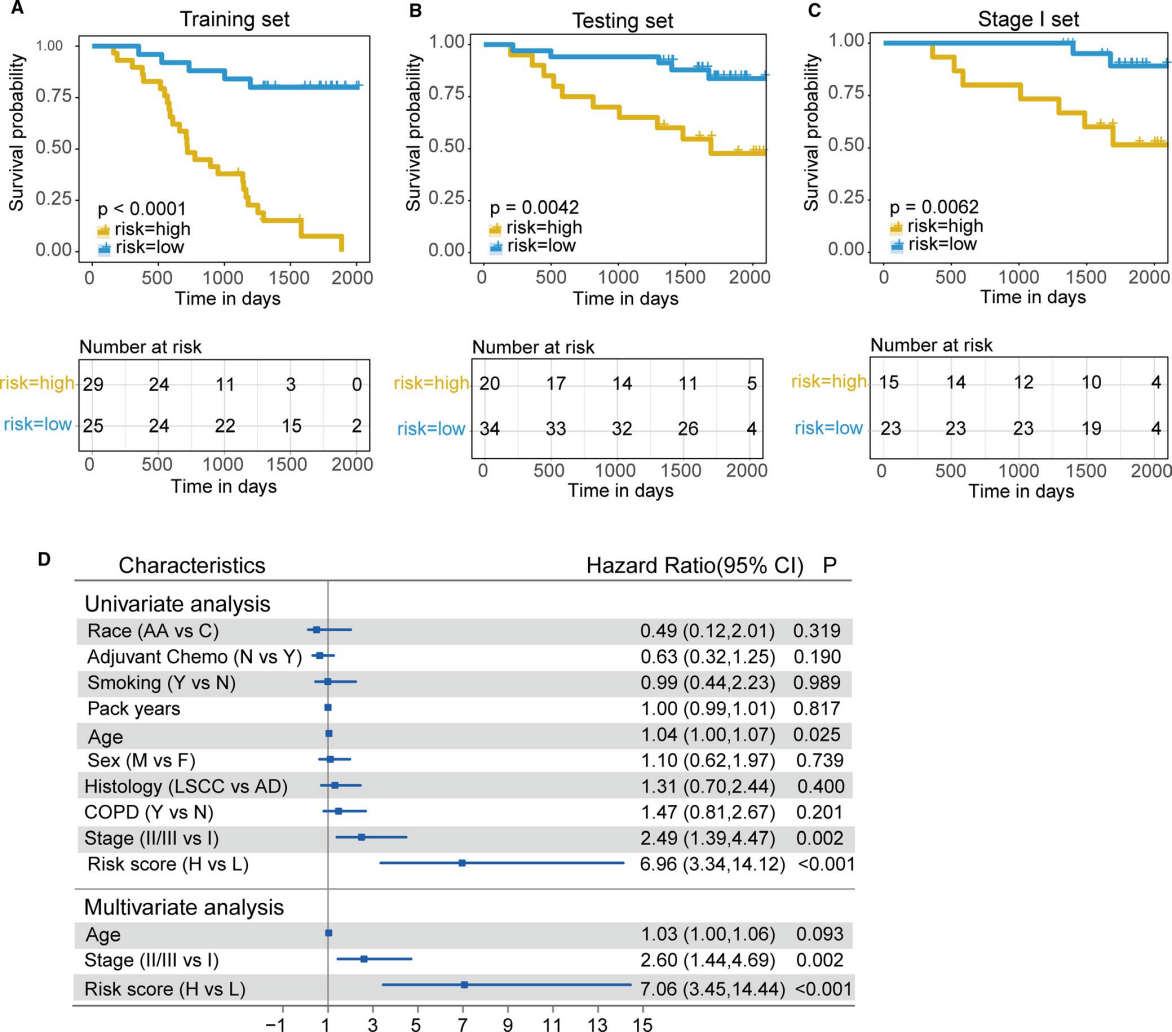
Predictive performance of the RS model. A, KM curves of the training set (54 samples) stratified by the RS cut‐off value. B, KM curves of the independent testing set (54 samples) stratified by the same cut‐off value. C, KM curves of the stage I subgroup (38 samples) from the test set. D, Univariate and multivariate regression analysis of the RS model and the other clinical characteristics regarding prognostic value

We calculated the RS for patients with expression values of the ten genes and corresponding regression coefficients and then divided the training set (n = 54) into high‐risk and low‐risk groups according to the optimum cut‐off value (cut‐off = −6.1; Figure S8C,D). Time‐dependent ROC analysis showed that the areas under the curve (AUCs) at 1, 2, 3 and 5 years were 0.897, 0.853, 0.813 and 0.911, respectively (Figure S9A). The high‐risk patients had poorer OS than patients with lower RS (log‐rank *P* < .0001, Figure 5A). The model showed good performance with the same cut‐off value in the testing set (n = 54, log‐rank *P* = .0042), even in patients with stage I disease (n = 38, *P* = .0062) (Figure 5B,C). The AUCs of ROC analysis at 1, 2, 3 and 5 years in the test set are shown in Figure S9B.

To determine whether the prognostic value of the RS was independent of other clinical characteristics, we conducted univariate and multivariate Cox regression analyses with the training set. The RS remained an independent prognostic indicator with the highest median hazard ratio (HR = 7.06, 95% confidence interval (CI) = 3.45‐14.44) for LC after adjusting for race, adjuvant chemotherapy, smoking status and annual amount, age, sex, histology, COPD status and pathologic stage (Figure 5D). Thus, the robustness of the RS for independently predicting LC patient OS was confirmed.

**FIGURE 6:**
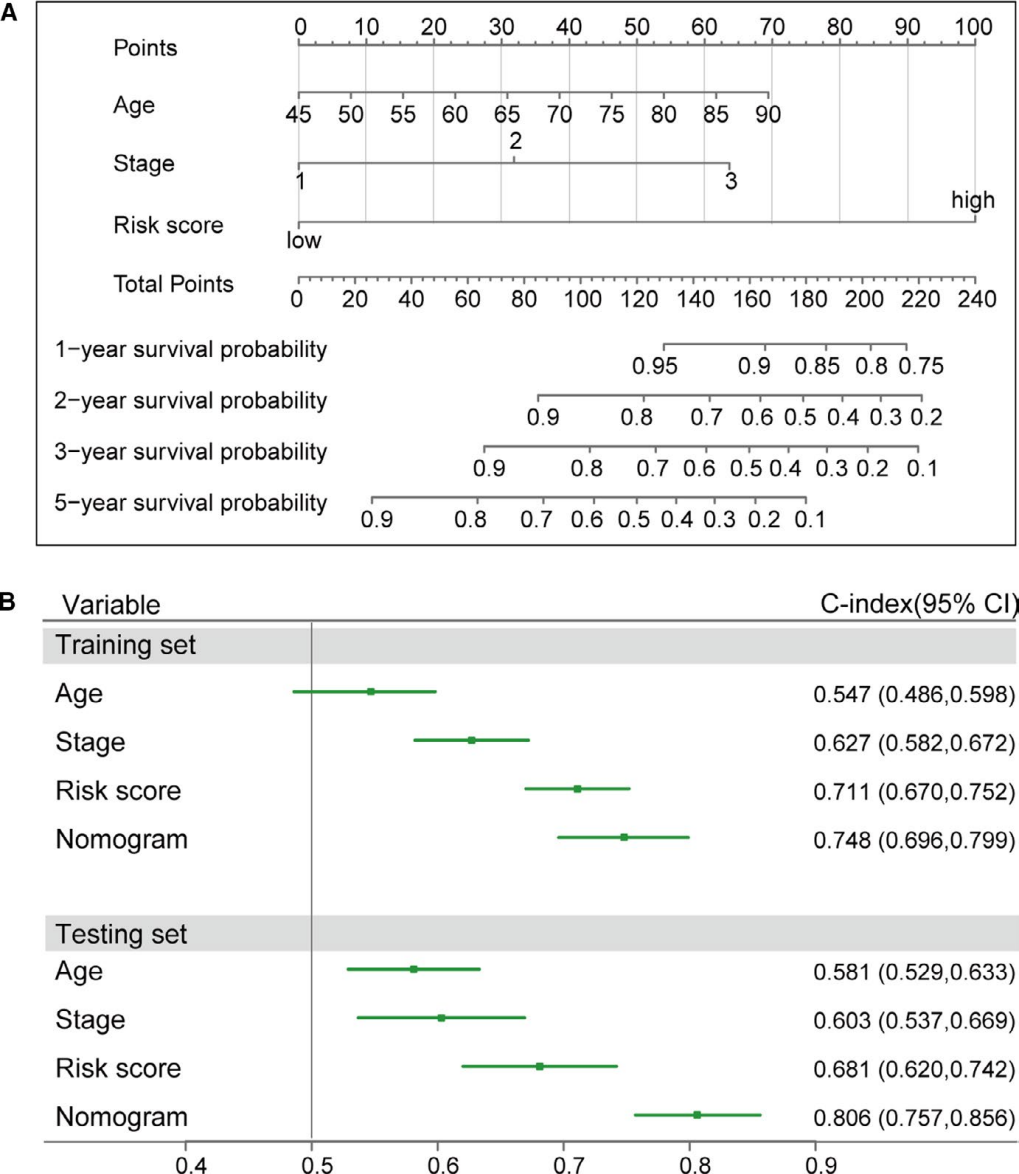
Nomogram model construction with RS, age and stage. A, Nomogram model for predicting the probability of 1‐, 2‐, 3‐ and 5‐year OS for LC patients. B, The prognostic performance was compared among the RS model, age, stage and the nomogram model by calculating the C‐index

We next constructed a nomogram that integrated the RS and conventional clinical traits in survival prediction, including age and stage, to provide a quantitative method for predicting the prognosis of LC patients (Figure 6A). In the nomogram, points of variables assigned by a point scale and the sum of all variable points were used as the total points to predict the survival probability of LC patients at 1, 2, 3 and 5 years. Remarkably, prediction with the RS prognosis model showed a higher C‐index than that with age or stage, and the nomogram model had a larger C‐index than the other three predictors in the training set and testing set (Figure 6B). Altogether, the RS model performed better than conventional clinical characteristics in survival prediction and constituted a quantitative method for predicting survival of LC patients, which was better than individual predictors.

## 4 DISCUSSION

Lung cancer (LC) is widely recognized as a highly heterogeneous disease that greatly threatens human health. Previously, many targeted studies have been conducted to comprehend the local immune status of LC lesions, but the understanding of variations in the peripheral immune system of the LC host is still limited.^30^, ^31^, ^32^ In this study, we characterized the host systemic immune response to LC and decrypted its heterogeneity. Two new LC subtypes were established based on the PBL transcriptome with different antitumour immune statuses and possible outcome differences. In addition, we constructed a RS prognostic model based on DEG signatures of the LC subtype with poor immune status to explore potential clinical application value.

The viewpoint that most tumours are systemic diseases that not only affect the organs of the lesion is increasingly accepted.^33^ Our study provides a comprehensive characterization of the systemic immune context in LC, which confirmed that the immune macroenvironment of LC has indeed undergone substantial changes at the PBL transcriptome level. An up‐regulated humoral immune response, regulation of lymphocyte activation and changes in immune cell proportions indicated a widespread impact of LC on host peripheral adaptive and innate immunity. Furthermore, enrichment of arachidonic acid metabolism and transcriptional misregulation in cancer supported that cancerous features may be captured from the peripheral blood transcriptome.^34^

In this study, we revealed substantial PBL transcriptome heterogeneity independent of histological type in LC patients. In other words, although different histological types have distinct origins, there seem to be no marked differences in influence on the peripheral immune system. Interestingly, similar findings were presented in research on breast cancer, and whether this observation represents a pan‐cancer phenomenon needs additional study to verify.^35^, ^36^, ^37^ Additionally, the heterogeneity seems to be related to stage in our study, raising the possibility that the PBL transcription profile can partly reflect the tumour burden of LC patients.

We tried to uncover the underlying biological mechanisms and significance of the heterogeneity. For LC1, one possible explanation for the marked up‐regulation of cell cycle–related pathways is that the proliferation of lymphocytes, especially CD8 T cells, is stimulated via antigen presentation by dendritic cells (DCs), thereby producing effector cells and enhancing cell immunity against tumour cells. Important effector T cells, cytotoxic T lymphocytes (CTLs) are derived from the clonal proliferation of CD8 CTL precursor cells and kill tumour cells by releasing granules or inducing FasL‐mediated apoptosis.^38^, ^39^ Analogously, NK cells perform broad‐spectrum antitumour functions employing similar cytotoxicity activities.^40^ Increased NK cell abundance and enhanced cytotoxicity in LC1 mean more NK cells were available for recruitment to exert antitumour cytotoxicity. As the only cells that lack a pro‐tumorigenic role, NK cells with a high proportion in peripheral blood or infiltration in tumour tissue often indicate a favourable outcome in cancer patients.^41^ Moreover, the high expression of stimulatory IMs may play an important role in the regulation of antitumour immunity in LC1. For example, CXCL9 mediates the trafficking of CD8 T cells, and CD70 is involved in the activation of CD8 T cells.^39^, ^42^

For LC2, we first observed activation of blood coagulation. Previous findings suggest that activation of blood clotting leads to fibrin deposition around the tumour, which can build up a provisional matrix to sustain and boost angiogenesis and induce the cellular responses of adhesion, proliferation and migration of tumour cells.^43^, ^44^ On the other hand, a higher neutrophil proportion and cytokine‐cytokine receptor interaction, regarded as crucial aspects of inflammation, were prominent in LC2. A growing body of evidence shows that inflammation promotes tumorigenesis and cancer progression.^45^ As the first responders to inflammation, active neutrophils release several soluble neutrophil granule proteins that can promote tumour progression by inducing tumour cell proliferation, stimulating angiogenesis and disabling T cell–dependent antitumour immunity.^46^, ^47^ Neutrophils facilitate the metastasis of circulating tumour cells, and cancer‐associated thrombosis by NETosis is considered to be an important factor in tumour progression.^48^ Cytokines are a double‐edged sword for carcinoma progression that are produced by host stromal cells and immune cells, either in response to molecules secreted by cancer cells or as part of the inflammation that frequently accompanies tumour growth. Through activating various downstream effectors, cytokines control the immune and inflammatory milieu to either favour antitumour immunity or enhance tumour progression and have direct effects on tumour cell growth and survival. For example, the Th1‐type cytokine IFN‐γ participates in the antitumour immune process, while TGF‐β and IL13 have the opposite action.^49^, ^50^, ^51^ In addition, the high expression levels of inhibitory immune checkpoint factors and low expression levels of NK cell activation receptors in LC2 demonstrate restrained lymphocyte activation and depletion of NK cells.^29^, ^52^ In general, the peripheral immunity of LC2 presents an inflammatory status, and antitumour immunity may be suppressed.

Considering the PBL transcriptome characteristics, we measured the prognostic relevance of DEG signatures derived from LC2. In terms of the ten signatures, in LC2, four risk factors were highly expressed (HR > 1; *CEACAM6*, *CEACAM1*, *HK3* and *SLC36A1*), and four protective factors showed low expression levels (HR < 1; *NR3C2*, *RLN2*, *TRPC1* and *FXYD7*), suggesting their potential important roles in the macroenvironment of LC among tumorigenesis and tumour progression (Figure S8F). The risk factors may be related to immunosuppression and inflammation. For instance, CEACAMs mediate cell adherence and transcellular transcytosis, resulting in the suppression of immune cell activities, as predictors in LC.^53^, ^54^ *HK3* correlated positively with inflammatory activities and multiple immune checkpoints.^55^ SLC36A1 may be involved in cancer metabolism.^56^ As for the protective factors, they may play a role in immune activation and anti‐inflammatory. *NR3C2* is considered to be a tumour suppressor and may be correlated with T cell activation.^57^ It is traditionally believed that M2 macrophages are generally associated with immunosuppression and tumour metastasis. TRPC1 and MSR1 can induce M2 to polarize into M1 macrophages.^58^, ^59^ RLN2 has anti‐inflammatory properties.^60^ However, the role of *FXYD7* and *HCG27* in the immune response is unclear.

The predictive ability of the RS model developed from PBMC data was validated even for patients with the same stage, proving the reliability of signature gene selection. The good performance of the prognostic model, especially the prognostic prediction for early‐stage LC patients, may help improve follow‐up treatment after surgical resection. More meaningfully, the RS contributed to constructing a comprehensive quantitative method for survival prediction, which performed better than existing clinical prognostic indicators.

We recognize limitations in our study. Due to the limitation of insufficient clinical data, we cannot demonstrate the potential clinical value of the heterogeneity between LC subsets directly in our data set. Moreover, both the heterogeneity of the PBL transcriptome and the universality of the RS prognostic model still require further validation with multicentre and larger scale studies.

In summary, to our knowledge, this study is the first to cover multiple LC histological types and stages and to target the peripheral blood transcriptome based on RNA‐Seq. We characterized the PBL transcriptional profiles and interpreted the heterogeneity of peripheral immunity in LC hosts for the first time. A robust RS prognostic model, as a non‐invasive and unbiased method to predict LC patient survival, was established and may help guide clinical treatment in the future.

## CONFLICT OF INTEREST

No conflicts of interest are declared.

## AUTHOR CONTRIBUTIONS

**Qi Zhang:** Formal analysis (lead); Methodology (lead); Resources (lead); Visualization (lead); Writing‐original draft (lead). **Manchao Kuang:** Data curation (lead); Methodology (equal); Validation (equal). **Haiyin An:** Data curation (equal). **Yajing Zhang:** Data curation (equal). **Kai Zhang:** Resources (equal). **Lin Feng:** Supervision (lead); Validation (equal); Writing‐review & editing (lead). **Lei Zhang:** Project administration (equal); Resources (equal). **Shujun Cheng:** Conceptualization (lead); Project administration (equal).

## Supporting information

Fig S1‐S9

Table S1‐S4

Supplementary Material

File S1

File S2

File S3

File S4

## Data Availability

The raw RNA‐Seq data for this study are deposited in the Genome Sequence Archive at the BIG Data Center, Beijing Institute of Genomics (BIG), Chinese Academy of Sciences, under the accession number HRA000350 [http://bigd.big.ac.cn/gsa‐human].

## References

[1] Bray F, Ferlay J, Soerjomataram I, Siegel RL, Torre LA, Jemal A. Global cancer statistics 2018: GLOBOCAN estimates of incidence and mortality worldwide for 36 cancers in 185 countries. CA Cancer J Clin. 2018;68:394‐424. 30207593 10.3322/caac.21492

[2] Altorki NK, Markowitz GJ, Gao D, et al. The lung microenvironment: an important regulator of tumour growth and metastasis. Nat Rev Cancer. 2019;19:9‐31. 30532012 10.1038/s41568-018-0081-9 PMC6749995

[3] Li B, Cui Y, Diehn M, Li R. Development and validation of an individualized immune prognostic signature in early‐stage nonsquamous non‐small cell lung cancer. JAMA Oncol. 2017;3:1529‐1537. 28687838 10.1001/jamaoncol.2017.1609 PMC5710196

[4] Forde PM, Chaft JE, Smith KN, et al. Neoadjuvant PD‐1 blockade in resectable lung cancer. N Engl J Med. 2018;378:1976‐1986. 29658848 10.1056/NEJMoa1716078 PMC6223617

[5] Hirsch FR, Scagliotti GV, Mulshine JL, et al. Lung cancer: current therapies and new targeted treatments. Lancet. 2017;389:299‐311. 27574741 10.1016/S0140-6736(16)30958-8

[6] Song L, Ma S, Chen L, Miao L, Tao M, Liu H. Long‐term prognostic significance of interleukin‐17‐producing T cells in patients with non‐small cell lung cancer. Cancer Sci. 2019;110:2100‐2109. 31100180 10.1111/cas.14068 PMC6609818

[7] Hong Y, Choi HM, Cheong HS, Shin HD, Choi CM, Kim WJ. Epigenome‐wide association analysis of differentially methylated signals in blood samples of patients with non‐small‐cell lung cancer. J Clin Med. 2019;8:1307. 10.3390/jcm8091307 PMC6780065 31450665

[8] Ros‐Mazurczyk M, Jelonek K, Marczyk M, et al. Serum lipid profile discriminates patients with early lung cancer from healthy controls. Lung Cancer. 2017;112:69‐74. 29191603 10.1016/j.lungcan.2017.07.036

[9] Rami‐Porta R, Asamura H, Travis WD, Rusch VW. Lung cancer ‐ major changes in the American Joint Committee on Cancer eighth edition cancer staging manual. CA Cancer J Clin. 2017;67(2):138‐155. 28140453 10.3322/caac.21390

[10] Li B, Zhang B, Wang X, et al. Expression signature, prognosis value, and immune characteristics of Siglec‐15 identified by pan‐cancer analysis. Oncoimmunology. 2020;9:1807291. 32939323 10.1080/2162402X.2020.1807291 PMC7480813

[11] Zhang C, Zhang G, Sun N, et al. Comprehensive molecular analyses of a TNF family‐based signature with regard to prognosis, immune features, and biomarkers for immunotherapy in lung adenocarcinoma. EBioMedicine. 2020;59:102959. 32853987 10.1016/j.ebiom.2020.102959 PMC7452643

[12] Snyder A, Nathanson T, Funt SA, et al. Contribution of systemic and somatic factors to clinical response and resistance to PD‐L1 blockade in urothelial cancer: an exploratory multi‐omic analysis. PLoS Medicine. 2017;14:e1002309. 28552987 10.1371/journal.pmed.1002309 PMC5446110

[13] Saffari A, Arno M, Nasser E, et al. RNA sequencing of identical twins discordant for autism reveals blood‐based signatures implicating immune and transcriptional dysregulation. Mol Autism. 2019;10:38. 31719968 10.1186/s13229-019-0285-1 PMC6839145

[14] World Medical Association . World Medical Association Declaration of Helsinki: ethical principles for medical research involving human subjects. JAMA. 2013;310:2191‐2194. 24141714 10.1001/jama.2013.281053

[15] Love MI, Huber W, Anders S. Moderated estimation of fold change and dispersion for RNA‐seq data with DESeq2. Genome Biol. 2014;15:550. 25516281 10.1186/s13059-014-0550-8 PMC4302049

[16] Yu G, Wang LG, Han Y, He QY. clusterProfiler: an R package for comparing biological themes among gene clusters. OMICS. 2012;16:284‐287. 22455463 10.1089/omi.2011.0118 PMC3339379

[17] Walter W, Sanchez‐Cabo F, Ricote M. GOplot: an R package for visually combining expression data with functional analysis. Bioinformatics. 2015;31:2912‐2914. 25964631 10.1093/bioinformatics/btv300

[18] Krijthe JH. Rtsne: T‐distributed stochastic neighbor embedding using a barnes‐hut implementation. 2015. https://github.com/jkrijthe/Rtsne,R package version 0.13.

[19] Pankla R, Buddhisa S, Berry M, et al. Genomic transcriptional profiling identifies a candidate blood biomarker signature for the diagnosis of septicemic melioidosis. Genome Biol. 2009;10:R127. 19903332 10.1186/gb-2009-10-11-r127 PMC3091321

[20] Weiner J 3rd, Domaszewska T. tmod: an R package for general and multivariate enrichment analysis. PeerJ Preprints. 2016.

[21] Langfelder P, Horvath S. WGCNA: an R package for weighted correlation network analysis. BMC Bioinformatics. 2008;9:559. 19114008 10.1186/1471-2105-9-559 PMC2631488

[22] Friedman J, Hastie T, Tibshirani R. Regularization paths for generalized linear models via coordinate descent. J Stat Softw. 2010;33:1‐22. 20808728 PMC2929880

[23] Avis IM, Jett M, Boyle T, et al. Growth control of lung cancer by interruption of 5‐lipoxygenase‐mediated growth factor signaling. J Clin Invest. 1996;97:806‐813. 8609238 10.1172/JCI118480 PMC507119

[24] Zhang L, Peng R, Sun Y, Wang J, Chong X, Zhang Z. Identification of key genes in non‐small cell lung cancer by bioinformatics analysis. PeerJ. 2019;7:e8215. 31844590 10.7717/peerj.8215 PMC6911687

[25] Riemann D, Cwikowski M, Turzer S, et al. Blood immune cell biomarkers in lung cancer. Clin Exp Immunol. 2019;195:179‐189. 30246868 10.1111/cei.13219 PMC6330648

[26] Sadasivam S, DeCaprio JA. The DREAM complex: master coordinator of cell cycle‐dependent gene expression. Nat Rev Cancer. 2013;13:585‐595. 23842645 10.1038/nrc3556 PMC3986830

[27] Dong P, Maddali MV, Srimani JK, et al. Division of labour between Myc and G1 cyclins in cell cycle commitment and pace control. Nat Commun. 2014;5:4750. 25175461 10.1038/ncomms5750 PMC4164785

[28] Thorsson V, Gibbs DL, Brown SD, et al. The immune landscape of cancer. Immunity. 2018;48:812‐830.e814. 29628290 10.1016/j.immuni.2018.03.023 PMC5982584

[29] Shi L, Li K, Guo Y, et al. Modulation of NKG2D, NKp46, and Ly49C/I facilitates natural killer cell‐mediated control of lung cancer. Proc Natl Acad Sci U S A. 2018;115:11808‐11813. 30381460 10.1073/pnas.1804931115 PMC6243255

[30] Guo X, Zhang Y, Zheng L, et al. Global characterization of T cells in non‐small‐cell lung cancer by single‐cell sequencing. Nat Med. 2018;24:978‐985. 29942094 10.1038/s41591-018-0045-3

[31] Karasaki T, Nagayama K, Kuwano H, et al. An immunogram for the cancer‐immunity cycle: towards personalized immunotherapy of lung cancer. J Thorac Oncol. 2017;12:791‐803. 28088513 10.1016/j.jtho.2017.01.005

[32] Tamborero D, Rubio‐Perez C, Muiños F, et al. A pan‐cancer landscape of interactions between solid tumors and infiltrating immune cell populations. Clin Cancer Res. 2018;24:3717‐3728. 29666300 10.1158/1078-0432.CCR-17-3509

[33] Allen BM, Hiam KJ, Burnett CE, et al. Systemic dysfunction and plasticity of the immune macroenvironment in cancer models. Nat Med. 2020;26:1125‐1134. 32451499 10.1038/s41591-020-0892-6 PMC7384250

[34] Umu SU, Langseth H, Keller A, et al. A 10‐year prediagnostic follow‐up study shows that serum RNA signals are highly dynamic in lung carcinogenesis. Mol Oncol. 2020;14:235‐247. 31851411 10.1002/1878-0261.12620 PMC6998662

[35] Ming W, Xie H, Hu Z, et al. Two distinct subtypes revealed in blood transcriptome of breast cancer patients with an unsupervised analysis. Front Oncol. 2019;9:985. 31632916 10.3389/fonc.2019.00985 PMC6779774

[36] Foulds GA, Vadakekolathu J, Abdel‐Fatah TMA, et al. Immune‐phenotyping and transcriptomic profiling of peripheral blood mononuclear cells from patients with breast cancer: identification of a 3 gene signature which predicts relapse of triple negative breast cancer. Front Immunol. 2018;9:2028. 30254632 10.3389/fimmu.2018.02028 PMC6141692

[37] Dumeaux V, Fjukstad B, Fjosne HE, et al. Interactions between the tumor and the blood systemic response of breast cancer patients. PLoS Comput Biol. 2017;13:e1005680. 28957325 10.1371/journal.pcbi.1005680 PMC5619688

[38] Kato T, Noma K, Ohara T, et al. Cancer‐associated fibroblasts affect intratumoral CD8(+) and FoxP3(+) T cells via IL6 in the tumor microenvironment. Clin Cancer Res. 2018;24:4820‐4833. 29921731 10.1158/1078-0432.CCR-18-0205

[39] Spranger S, Gajewski TF. Impact of oncogenic pathways on evasion of antitumour immune responses. Nat Rev Cancer. 2018;18:139‐147. 29326431 10.1038/nrc.2017.117 PMC6685071

[40] Chiossone L, Dumas PY, Vienne M, Vivier E. Natural killer cells and other innate lymphoid cells in cancer. Nat Rev Immunol. 2018;18:671‐688. 30209347 10.1038/s41577-018-0061-z

[41] Hoshikawa M, Aoki T, Matsushita H, et al. NK cell and IFN signatures are positive prognostic biomarkers for resectable pancreatic cancer. Biochem Biophys Res Commun. 2018;495:2058‐2065. 29253566 10.1016/j.bbrc.2017.12.083

[42] Borst J, Ahrends T, Babala N, Melief CJM, Kastenmuller W. CD4(+) T cell help in cancer immunology and immunotherapy. Nat Rev Immunol. 2018;18:635‐647. 30057419 10.1038/s41577-018-0044-0

[43] Mosesson MW. Fibrinogen and fibrin structure and functions. J Thromb Haemost. 2005;3:1894‐1904. 16102057 10.1111/j.1538-7836.2005.01365.x

[44] Palumbo JS, Kombrinck KW, Drew AF, et al. Fibrinogen is an important determinant of the metastatic potential of circulating tumor cells. Blood. 2000;96:3302‐3309. 11071621

[45] Greten FR, Grivennikov SI. Inflammation and cancer: triggers, mechanisms, and consequences. Immunity. 2019;51:27‐41. 31315034 10.1016/j.immuni.2019.06.025 PMC6831096

[46] Deryugina EI, Zajac E, Juncker‐Jensen A, Kupriyanova TA, Welter L, Quigley JP. Tissue‐infiltrating neutrophils constitute the major in vivo source of angiogenesis‐inducing MMP‐9 in the tumor microenvironment. Neoplasia. 2014;16:771‐788. 25379015 10.1016/j.neo.2014.08.013 PMC4212255

[47] Munder M, Schneider H, Luckner C, et al. Suppression of T‐cell functions by human granulocyte arginase. Blood. 2006;108:1627‐1634. 16709924 10.1182/blood-2006-11-010389

[48] Jorch SK, Kubes P. An emerging role for neutrophil extracellular traps in noninfectious disease. Nat Med. 2017;23:279‐287. 28267716 10.1038/nm.4294

[49] Budhu S, Schaer DA, Li Y, et al. Blockade of surface‐bound TGF‐beta on regulatory T cells abrogates suppression of effector T cell function in the tumor microenvironment. Sci Signal. 2017;10(494):eaak9702. 28851824 10.1126/scisignal.aak9702 PMC5851440

[50] Bartolome RA, Garcia‐Palmero I, Torres S, Lopez‐Lucendo M, Balyasnikova IV, Casal JI. IL13 receptor alpha2 signaling requires a scaffold protein, FAM120A, to activate the FAK and PI3K pathways in colon cancer metastasis. Cancer Res. 2015;75:2434‐2444. 25896327 10.1158/0008-5472.CAN-14-3650

[51] Alspach E, Lussier DM, Schreiber RD. Interferon gamma and its important roles in promoting and inhibiting spontaneous and therapeutic cancer immunity. Cold Spring Harb Perspect Biol. 2019;11(3):a028480. 29661791 10.1101/cshperspect.a028480 PMC6396335

[52] Park J‐J, Omiya R, Matsumura Y, et al. B7–H1/CD80 interaction is required for the induction and maintenance of peripheral T‐cell tolerance. Blood. 2010;116:1291‐1298. 20472828 10.1182/blood-2010-01-265975 PMC2938239

[53] Beauchemin N, Arabzadeh A. Carcinoembryonic antigen‐related cell adhesion molecules (CEACAMs) in cancer progression and metastasis. Cancer Metastasis Rev. 2013;32:643‐671. 23903773 10.1007/s10555-013-9444-6

[54] Laack E, Nikbakht H, Peters A, et al. Expression of CEACAM1 in adenocarcinoma of the lung: a factor of independent prognostic significance. J Clin Oncol. 2002;20:4279‐4284. 12409325 10.1200/JCO.2002.08.067

[55] Tuo Z, Zheng X, Zong Y, et al. HK3 is correlated with immune infiltrates and predicts response to immunotherapy in non‐small cell lung cancer. Clin Transl Med. 2020;10:319‐330. 32508023 10.1002/ctm2.6 PMC7240846

[56] Ögmundsdóttir MH, Heublein S, Kazi S, et al. Proton‐assisted amino acid transporter PAT1 complexes with Rag GTPases and activates TORC1 on late endosomal and lysosomal membranes. PLoS One. 2012;7:e36616. 22574197 10.1371/journal.pone.0036616 PMC3344915

[57] Lu J, Hu F, Zhou Y. NR3C2‐related transcriptome profile and clinical outcome in invasive breast carcinoma. Biomed Res Int. 2021;2021:9025481. 33564687 10.1155/2021/9025481 PMC7867450

[58] Nascimento Da Conceicao V, Sun Y, Zboril EK, De la Chapa JJ, Singh BB. Loss of Ca(2+) entry via Orai‐TRPC1 induces ER stress, initiating immune activation in macrophages. J Cell Sci. 2019;133(5):jcs237610. 31722977 10.1242/jcs.237610 PMC10682644

[59] Chen Z, Huang H, Wang Y, Zhan F, Quan Z. Identification of immune‐related genes MSR1 and TLR7 in relation to macrophage and Type‐2 T‐helper cells in osteosarcoma tumor micro‐environments as anti‐metastasis signatures. Front Mol Biosci. 2020;7:576298. 33381518 10.3389/fmolb.2020.576298 PMC7768026

[60] Bathgate RA, Halls ML, van der Westhuizen ET, Callander GE, Kocan M, Summers RJ. Relaxin family peptides and their receptors. Physiol Rev. 2013;93:405‐480. 23303914 10.1152/physrev.00001.2012

