# Supplementary material for: Peripheral blood transcriptome heterogeneity and prognostic potential in lung cancer revealed by RNA‐Seq
Source: J Cell Mol Med. 2021 Jul 21;25(17):8271–84. doi: 10.1111/jcmm.16773 (PMC8419186; doi:10.1111/jcmm.16773)
Supplement: Supplementary file 1 — Fig S1‐S9 [file JCMM-25-8271-s003.docx]

# Supplementary Figures

Figure S1


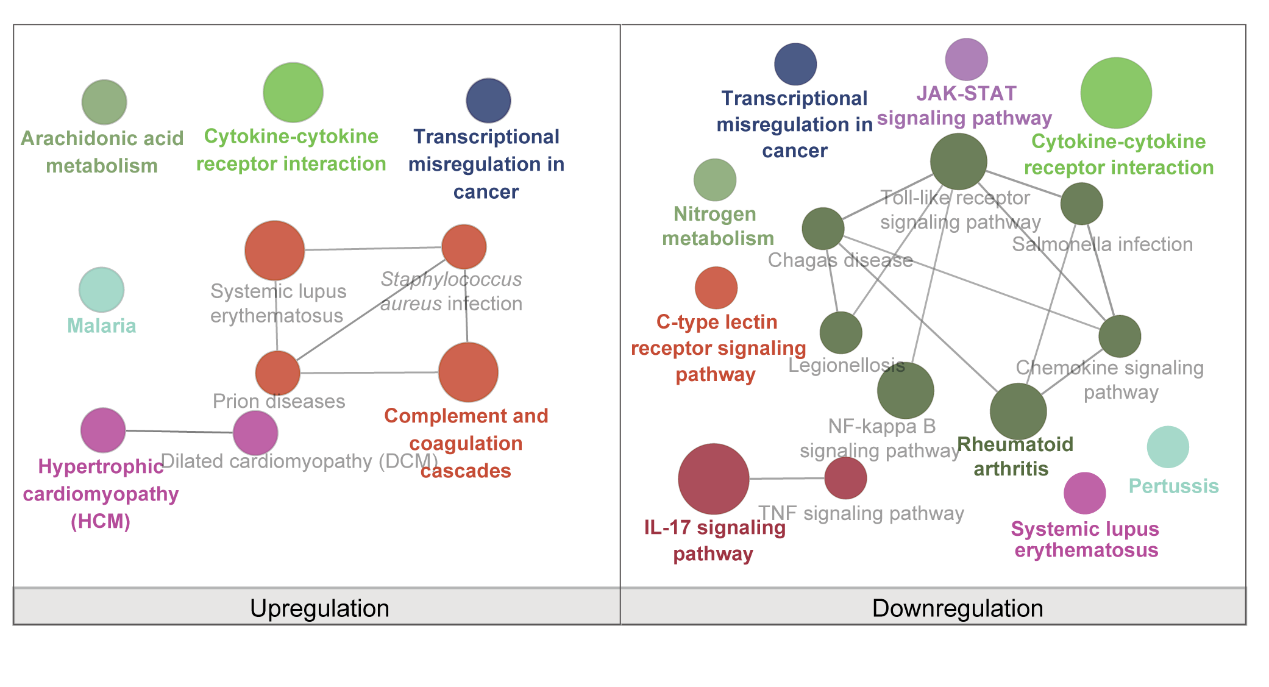


**Figure S1. KEGG** **pathway enrichment analysis with all DEGs (LC vs healthy).** Upregulated and downregulated pathways are shown by Cytoscape. The size of nodes indicates the significance level of the association of genes per pathway.

Figure S2


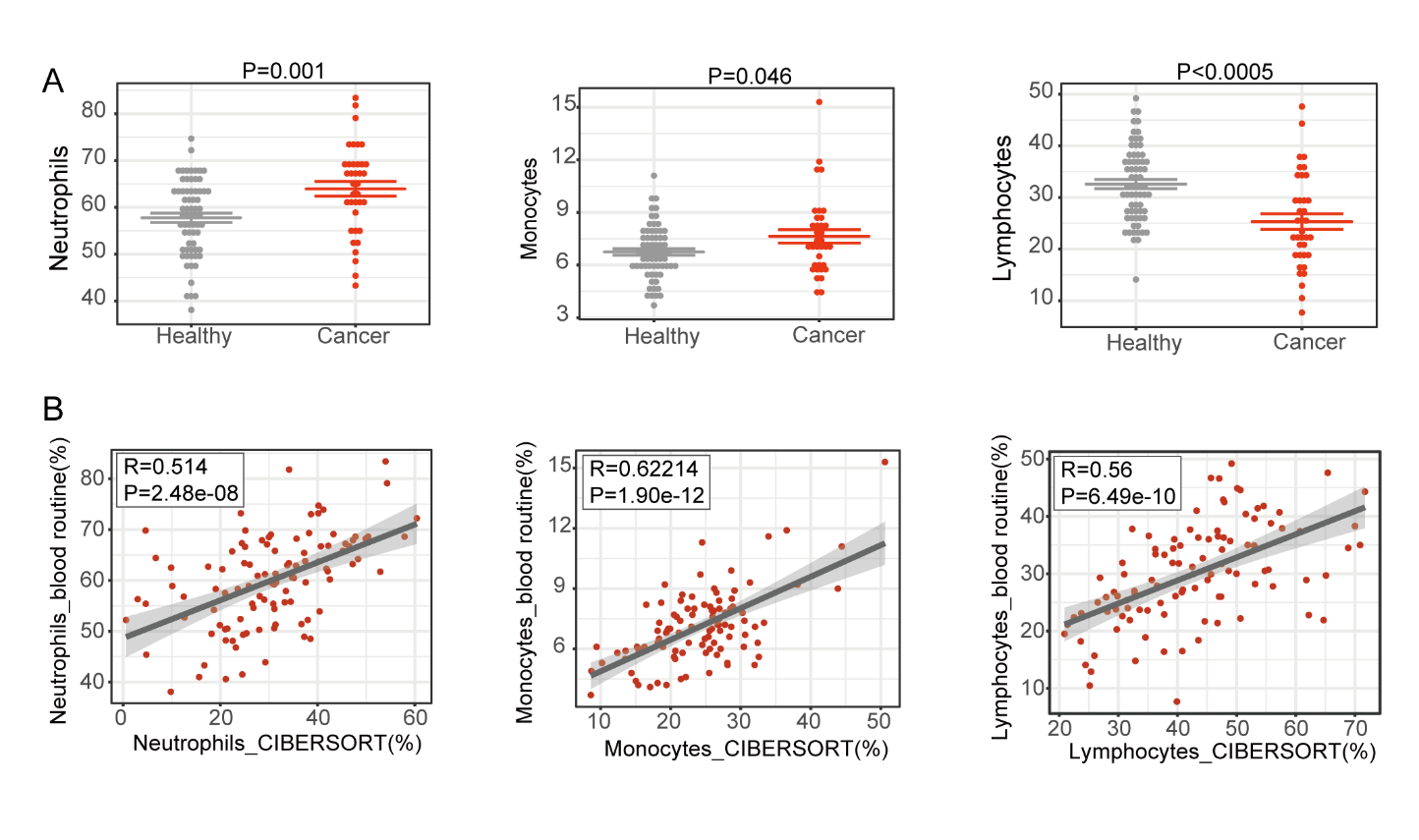


**Figure S2. Blood routine indicators of subjects.** (A) Comparation of neutrophils, monocytes, and lymphocytes between LC and healthy controls based on routine blood tests (Mann-Whitney U test). (B) Correlation between CIBERSORT analysis results and blood routine indicators of 67 healthy and 37 LC samples (Pearson's product-moment correlation test). Bars show mean ± SEM.

Figure S3


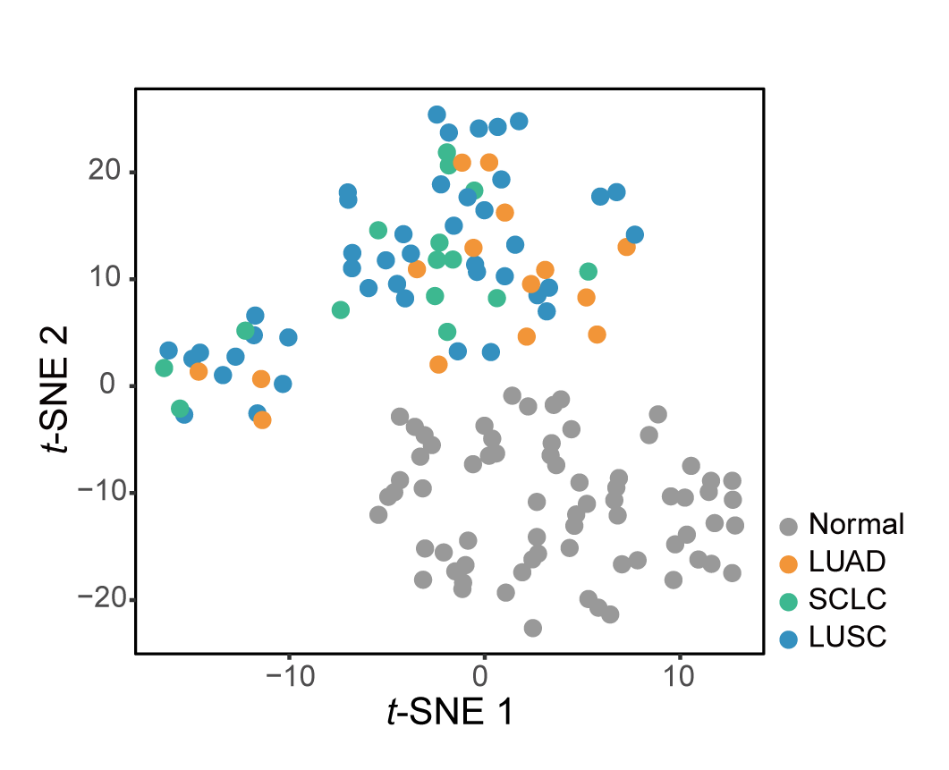


**Figure S3. t-SNE plot of all samples coloured by histological type (LUAD, lung adenocarcinoma; SCLC, small cell lung cancer; LUSC, lung squamous cell carcinoma).**

Figure S4


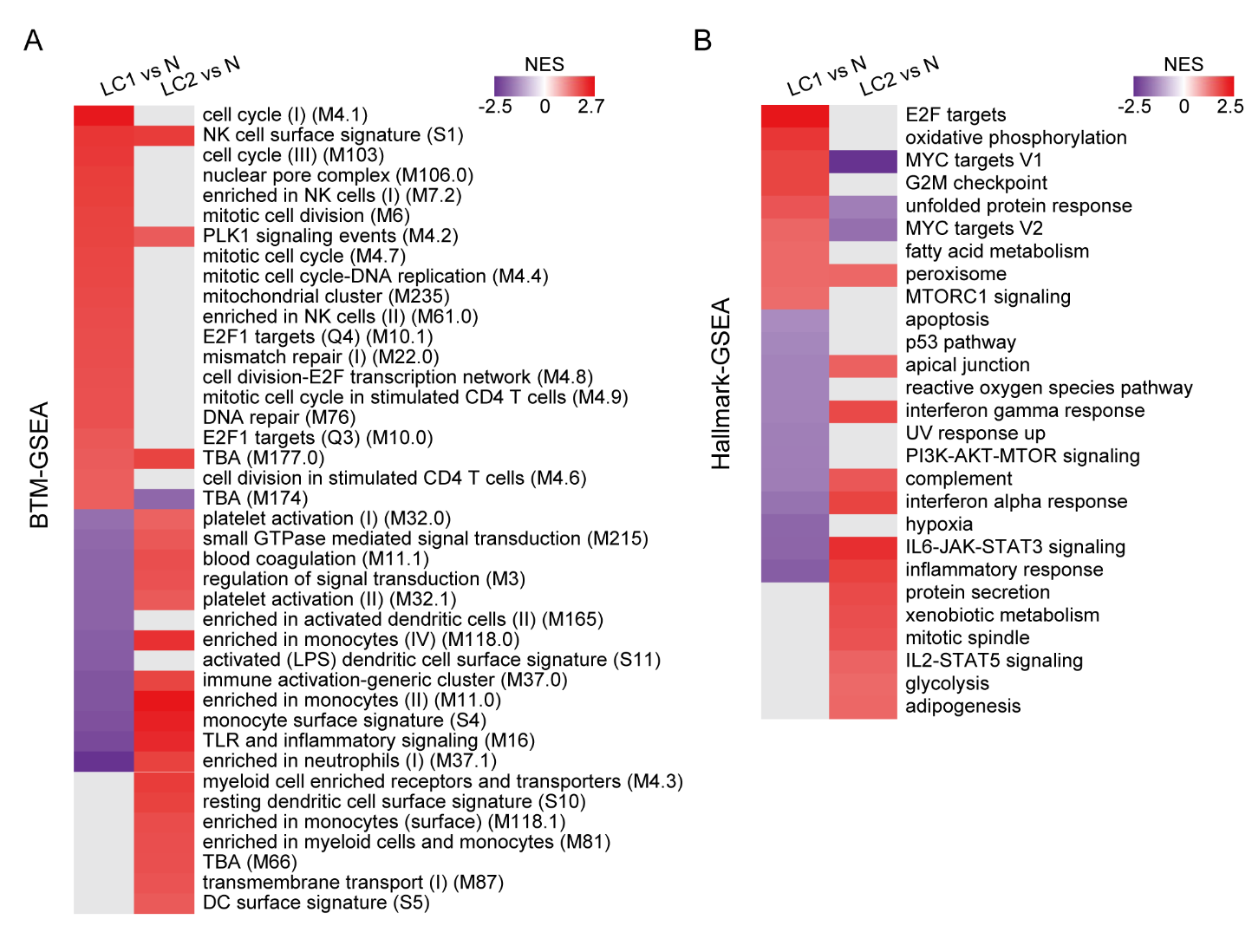


**Figure S4. GSEA of the PBL transcriptome of the two LC subtypes.**

(A) GSEA based on BTMs of the PBL transcriptome. (B) GSEA based on hallmark gene sets of the PBL transcriptome.

Figure S5


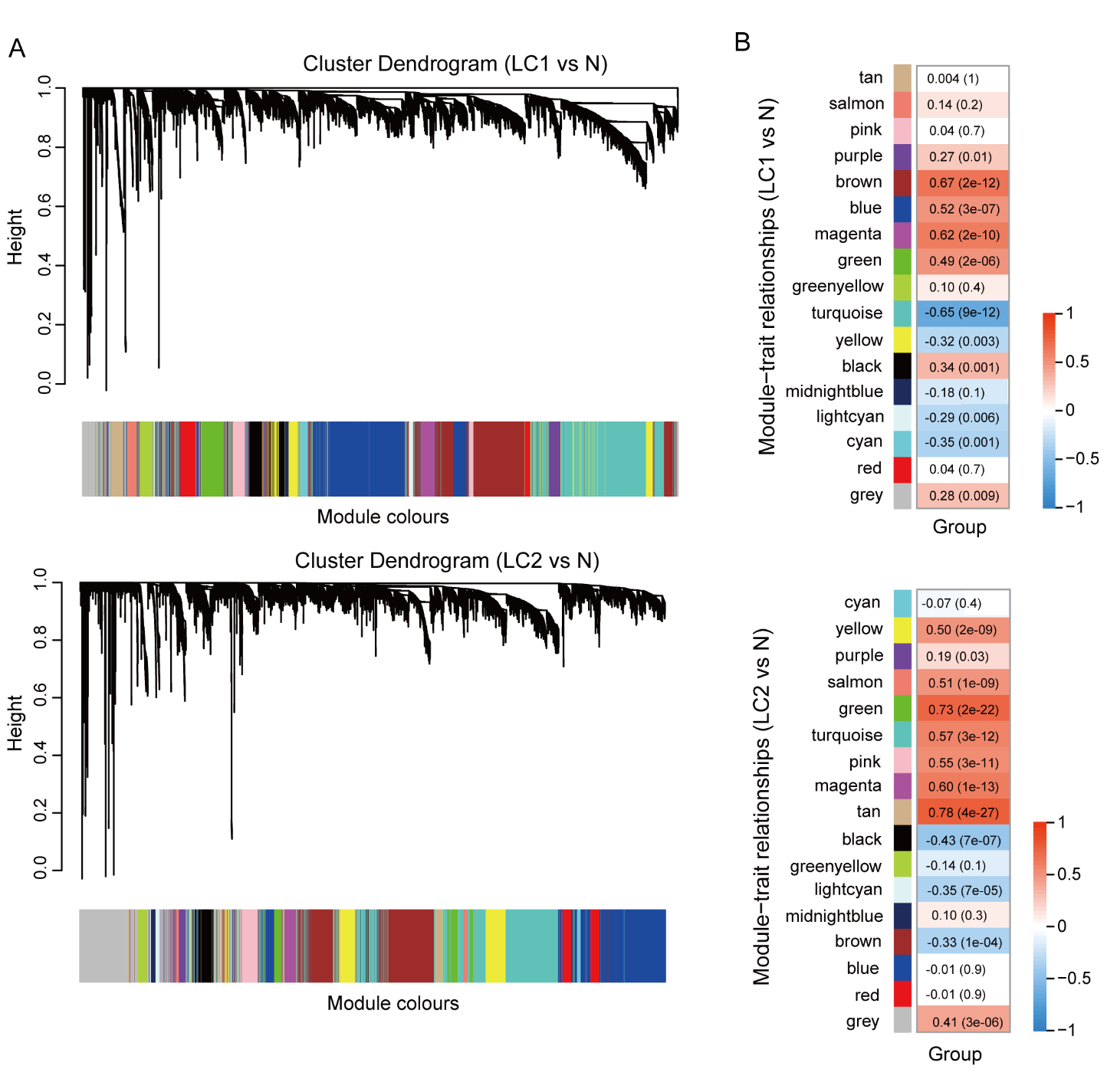


**Figure S5. Co-expression modules constructed by WGCNA.**

(A) Construction of the gene co-expression network for LC1 vs N and LC2 vs N. Clustering dendrograms of genes with differences based on topological overlap are displayed as different coloured modules. (B) Module-trait relationships in the LC1 and LC2 networks shown as heatmaps. Each row represents a module that contains a group of eigengenes, while the column represents sample group characteristics. Each cell is colour-coded by correlation according to the colour legend, and the p value is shown.

Figure S6


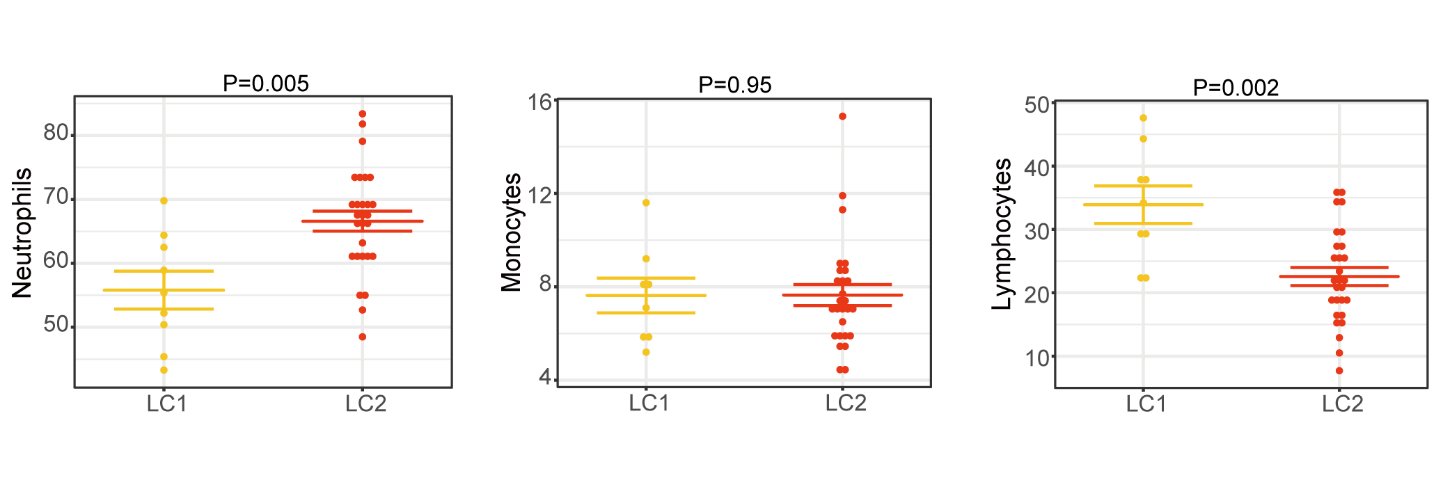


**Figure S6. Routine blood analysis of neutrophils, monocytes and lymphocytes between the two LC subtypes (Mann-Whitney U test).**

Figure S7


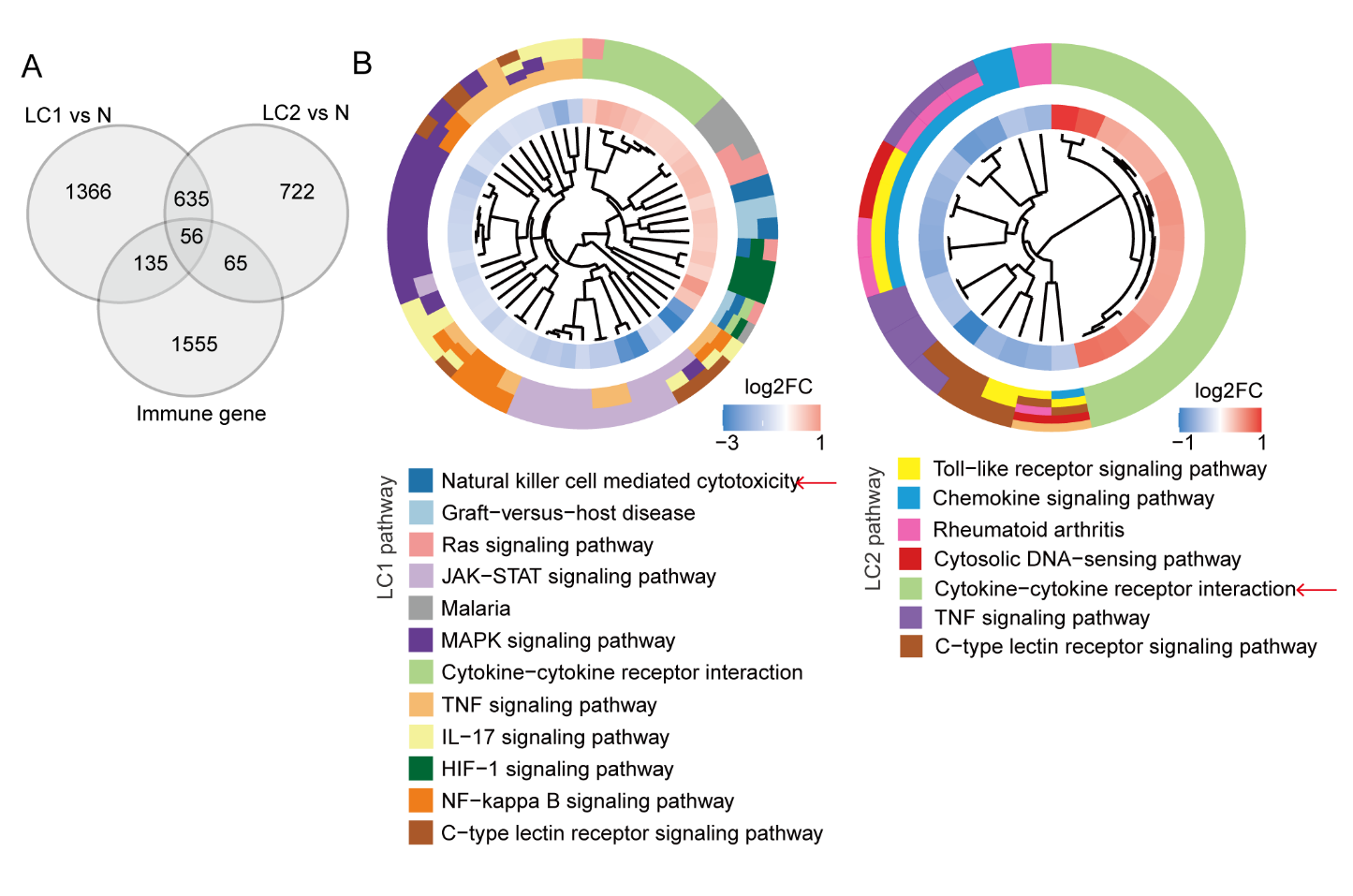
**Figure S7. KEGG pathway analysis of two gene panels identified in the LC subtypes independent of cell composition.**

(A) Venn diagram showing the overlap between immune genes and two groups of DEGs after correcting for the ratio of the proportions of myeloid cells to lymphocytes (FDR <0.05, |log2FC| ≥0.59). (B) KEGG pathway analysis of the overlapping immune-related DEGs after correction. Only the top five upregulated and downregulated pathways are shown in each group (FDR < 0.05). The inner circle shows log2FC values of corrected DEGs in enriched pathways.

Figure S8


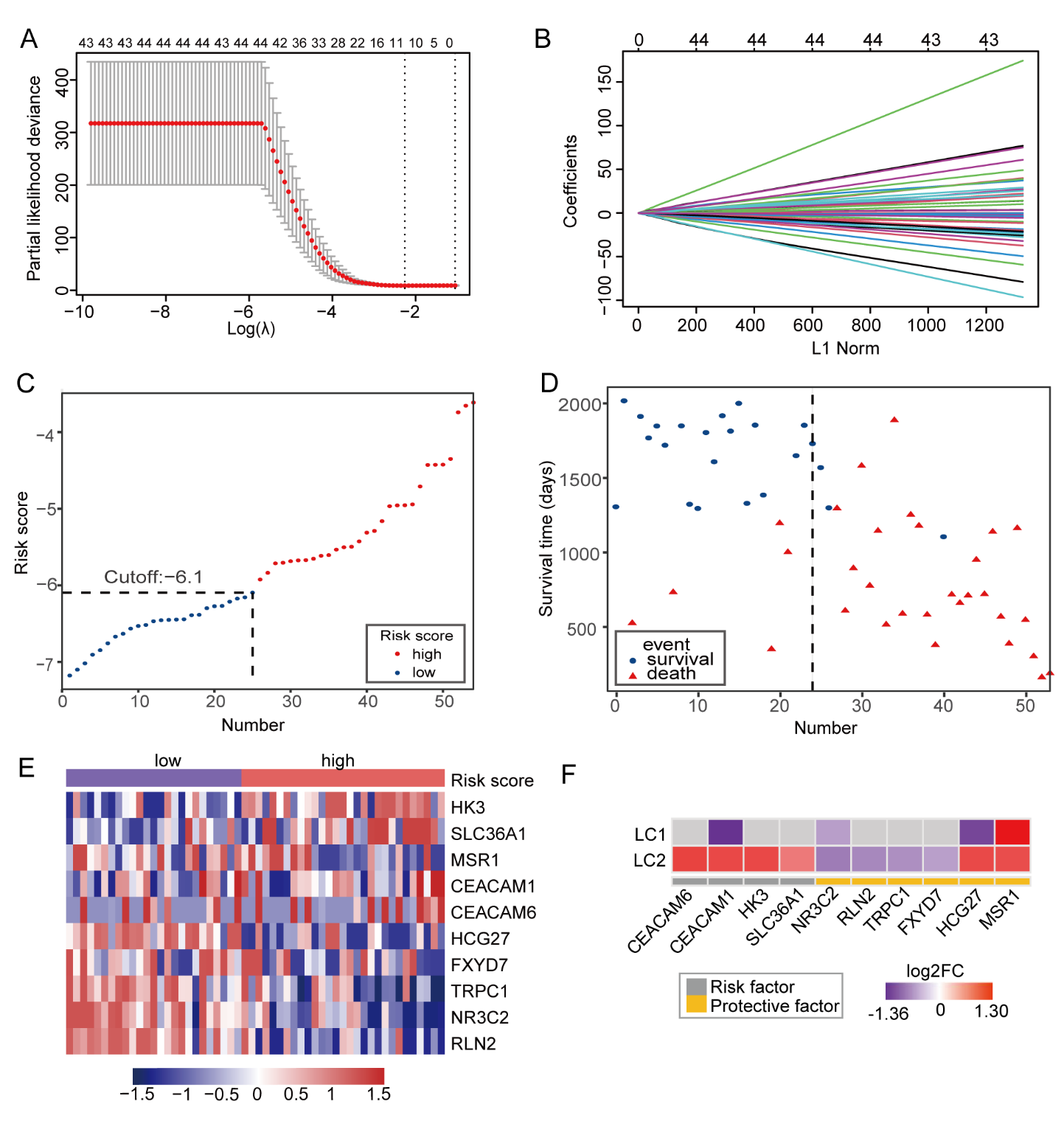


**Figure S8. Construction of the RS model for LC patients by LASSO Cox regression.**

(A) Partial likelihood deviance for the LASSO coefficient profiles. The x-axis represents the log value of the independent variable lambda, and the y-axis represents the coefficient of the independent variable. (B) LASSO coefficient profiles of each lambda. (C) Comparison of the RS of each subgroup in the training set. The horizontal axis represents the samples, and the vertical axis represents the RS. (D) Survival event distribution in the training set. The horizontal axis represents the samples, and the vertical axis represents OS. (E) Expression pattern of ten prognostic genes in the training set. (F) Expression pattern of the ten prognostic genes in LC1 and LC2. Coloured cells indicate DEGs (FDR<0.05), and grey cells indicate no significant difference.

Figure S9


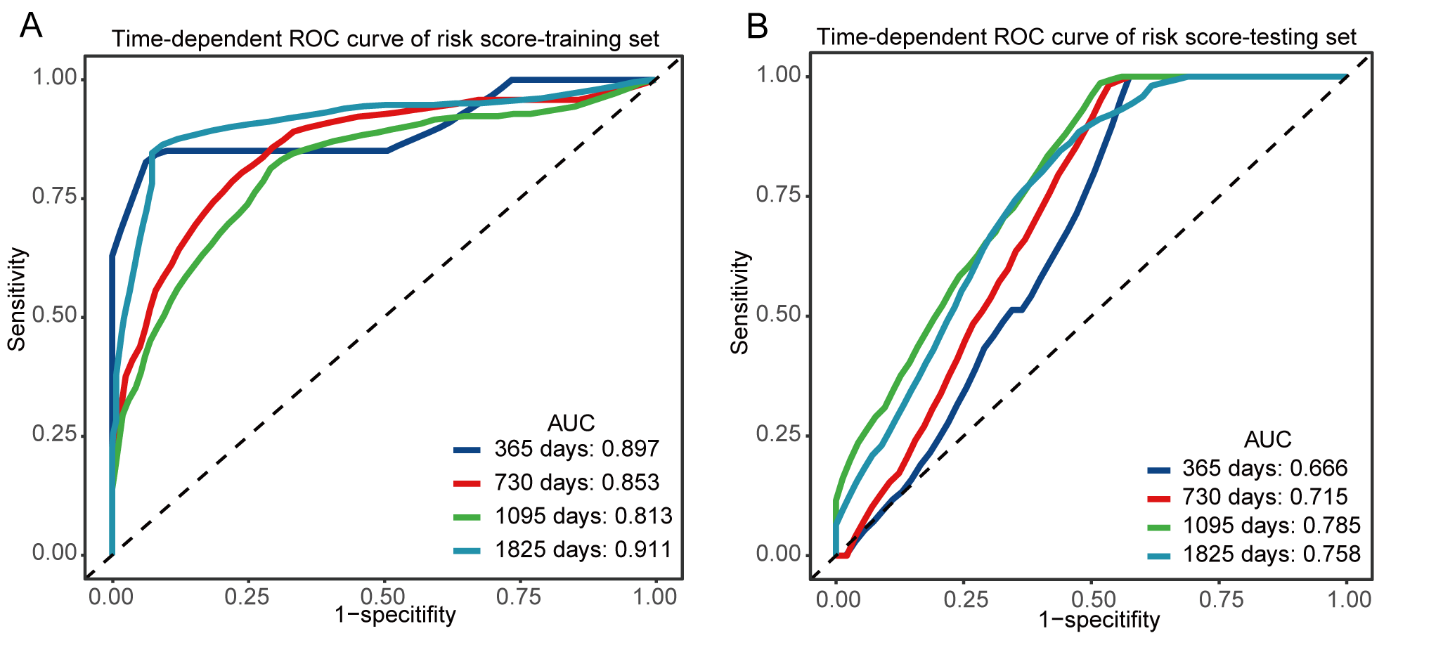


**Figure S9. Results for time-dependent ROC analysis of the RS at 1, 2, 3, and 5 years for the training set (A) and testing set (B).**
