# Supplementary material for: Peripheral blood transcriptome heterogeneity and prognostic potential in lung cancer revealed by RNA‐Seq
Source: J Cell Mol Med. 2021 Jul 21;25(17):8271–84. doi: 10.1111/jcmm.16773 (PMC8419186; doi:10.1111/jcmm.16773)
Supplement: Supplementary file 2 — Table S1‐S4 [file JCMM-25-8271-s007.docx]

# Supplementary Tables

**Table S1 Basic clinical characteristics of LC patients and healthy volunteers.**

|  | **LUAD (n=15)** | **LUSC (n=43)** | **SCLC (n=15)** | **H (n=69)** |
| --- | --- | --- | --- | --- |
| Age | | | | |
| Median (range) | 64 (32, 76) | 62 (42, 83) | 62 (44, 76) | 56 (48, 75) |
| Sex, N (%) | | | | |
| Male | 8 (53.33) | 43 (100.00) | 12 (80.00) | 38 (55.26) |
| Female | 7 (46.67) | 0 (0.00) | 3 (20.00) | 31 (44.74) |
| TNM stage, N (%) | | | | |
| I | 1 (6.67) | 4 (9.30) | 0 (0.00) | — |
| II | 4 (26.67) | 16 (37.21) | 1 (6.67) | — |
| III | 4 (26.67) | 21 (48.84) | 9 (60.00) | — |
| IV | 6 (40.00) | 2 (4.65) | 5 (33.33) | — |

*Note*: LUAD, lung adenocarcinoma; LUSC, lung squamous cell carcinoma; SCLC, small cell lung cancer; H, healthy

**Table S2** **Characteristics of the two LC patient subgroups.**

|  | **LC1 (n=17)** | **LC2 (n=56)** | **P value** |
| --- | --- | --- | --- |
| Stage | | | |
| I | 3 (17.65%) | 2 (3.57%) | 0.004^1^ |
| II | 9 (52.94%) | 12 (21.43%) |  |
| III | 5 (29.41%) | 29 (51.79%) |  |
| IV | 0 (0.00%) | 13 (23.21%) |  |
| Histology | | | |
| LUAD | 3 (17.65%) | 12 (21.43%) | 0.857^1^ |
| LUSC | 11 (64.70%) | 32 (57.14%) |  |
| SCLC | 3 (17.65%) | 12 (21.43%) |  |
| Sex | | | |
| M | 15 (88.24%) | 48 (85.71%) | 0.791^3^ |
| F | 2 (11.76%) | 8 (14.29%) |  |
| Age | | | |
| Median | 63 (51, 85) | 62 (32, 76) | 0.267^2^ |
| COPD | | | |
| Y | 2 (11.76%) | 9 (16.07%) |  |
| N | 3 (17.65%) | 18 (32.14%) | 0.406^1^ |
| UN | 12 (70.59%) | 29 (51.79%) |  |
| Smoking | | | |
| Y | 13 (76.47%) | 47 (83.93%) | 0.732^3^ |
| N | 4 (23.53%) | 9 (16.07%) |  |

*Note*: LC1: lung cancer set 1; LC2: lung cancer set 2

NS: not significant (P>0.05)

^1^ Fisher’s exact Chi-square test

^2^ Unpaired Student's t‐test

^3^ Chi-square test with Yates correction for continuity

**Table S3 Characteristics of LC patients (GSE13255).**

| **Characteristics** | **Number of cases (%)** |
| --- | --- |
| Smoking |  |
| N | 6 (5.56) |
| Q | 87 (80.56) |
| S | 15 (13.89) |
| Race |  |
| AA | 9 (8.33) |
| C | 99 (91.67) |
| Sex |  |
| F | 55 (50.93) |
| M | 53 (49.07) |
| COPD |  |
| n | 54 (50) |
| un | 4 (3.70) |
| y | 50 (46.30) |
| Histology |  |
| AD | 67 (62.04) |
| LSCC | 34 (31.48) |
| NSCLC | 7 (6.48) |
| Age |  |
| ≤68 | 55 (50.93) |
| >68 | 53 (49.07) |
| Pack/year |  |
| ≤40 | 56 (51.85) |
| >40 | 52 (48.15) |
| Stage |  |
| I | 66 (61.11) |
| II | 14 (12.96) |
| III | 28 (25.93) |

**Table S4** **The 10 genes of the RS prediction model.**

| **Gene ID** | **Type** | **Univariate analysis** | | | **LASSO coefficient** |
| --- | --- | --- | --- | --- | --- |
|  |  | **HR** | **95% CI** | **P value** |  |
| NR3C2 | mRNA | 0.36 | 0.21-0.61 | 0.00017 | - 0.70761344 |
| TRPC1 | mRNA | 0.48 | 0.31-0.75 | 0.00130 | - 0.08267232 |
| HCG27 | ncRNA | 0.58 | 0.41-0.83 | 0.00260 | - 0.47834666 |
| FXYD7 | mRNA | 0.58 | 0.35-0.97 | 0.03600 | - 0.07974512 |
| RLN2 | mRNA | 0.58 | 0.41-0.80 | 0.00100 | - 0.12672934 |
| MSR1 | mRNA | 0.60 | 0.41-0.86 | 0.00590 | - 0.40941127 |
| CEACAM6 | mRNA | 1.20 | 1.00-1.40 | 0.04300 | 0.12027717 |
| CEACAM1 | mRNA | 1.40 | 1.10-1.70 | 0.01600 | 0.10972726 |
| SLC36A1 | mRNA | 2.70 | 1.40-5.20 | 0.00300 | 0.07896976 |
| HK3 | mRNA | 3.20 | 1.60-6.50 | 0.00120 | 0.33021614 |

*Note:* HR: hazard ratio.
