# Supplementary material for: Peripheral blood transcriptome heterogeneity and prognostic potential in lung cancer revealed by RNA‐Seq
Source: J Cell Mol Med. 2021 Jul 21;25(17):8271–84. doi: 10.1111/jcmm.16773 (PMC8419186; doi:10.1111/jcmm.16773)
Supplement: Supplementary file 3 — Supplementary Material [file JCMM-25-8271-s001.docx]

**Supplementary methods**

**Collection of specimens**

We recruited 69 healthy individuals who received a physical examination and 73 histopathologically confirmed LC patients at the Cancer Institute Hospital of the Chinese Academy of Medical Sciences (CAMS) from 2016 to 2017. The inclusion criteria of the patients included previously untreated primary LC without inflammatory, autoimmune, infectious, or other systemic diseases. Healthy subjects, in addition to meeting the above conditions, could not have benign tumours or abnormal serology tumour markers. Four millilitres of fresh peripheral blood from each enrolled individual was collected in K2 EDTA vacuum tubes (BD Biosciences, Franklin Lakes, NJ, USA; cat. no. 367844) before clinical treatment.

**Generation and normalization of RNA sequencing data**

We isolated blood cells from peripheral whole blood by centrifugation and then lysed red blood cells using erythrocyte lysis buffer (Qiagen, Hilden, Germany) to finally obtain leukocytes within 6 hours after collecting samples. Afterwards, total RNA extraction was conducted with TRIzol solution (Invitrogen, Carlsbad, CA, USA). The integrity of RNA samples with OD260/280≥1.9 detected by a NanoDrop2000 spectrophotometer (Thermo Scientific) was further corroborated using a Bioanalyzer 2100 (Agilent, USA). Eventually, eligible libraries prepared from qualified samples using an NEBNext^®^ Ultra™ RNA Library Prep Kit (New England Biolabs, Ipswich, MA, UK) were pooled and sequenced as 150 bp paired-end reads on the Illumina HiSeq 4000 platform.

Raw reads were processed with the Cutadapt tool and were trimmed by using Sickle software (<https://github.com/najoshi/sickle>).^1^ Then, clean reads were mapped to the human reference genome (GRCh38) by Salmon.^2^ Transcript abundances were summarized at the gene level with tximport and were normalized based on transcripts-per-million (TPM).^3^ After filtering lowly expressed genes (genes with zero TPM values in more than 10% of all samples), a total of 19760 genes were included in all downstream analyses to maintain consistency. The TPM values of the remaining genes were transformed by log2 (TPM+1).

**Method for determining the optimal threshold of riskscore**

In order to determine the optimal cutoff value, we set the overall survival to 5 years and used Kaplan-Meier (KM) method to construct the receiver operating characteristic (ROC) curve by survivalROC package. The points on the curve represent different cutoff values (risk scores), and the x-axis and y-axis represent the “1-specitifity” and “sensitivity” corresponding to different cutoff values, respectively. We computed Youden Index (Youden Index = Sensitivity + Specificity -1) on the basis of the ROC curve. We chose the point with the largest Youden Index as the optimal cutoff value (-6.1), and divided the patients into high-risk and low-risk groups.

**Reference**

1 MARTIN M. Cutadapt removes adapte sequences from high-throughput sequencing reads. *EMBnetjournal*. 2011; 17: 10–12.

2 Patro R, Duggal G, Love MI, Irizarry RA, Kingsford C. Salmon provides fast and bias-aware quantification of transcript expression. *Nat Methods*. 2017; 14: 417-419.

3 Soneson C, Love MI, Robinson MD. Differential analyses for RNA-seq: transcript-level estimates improve gene-level inferences. *F1000Res*. 2015; 4: 1521.
